# NEDD4-binding protein 1 suppresses hepatitis B virus replication by regulating viral RNAs

**DOI:** 10.1099/jgv.0.002082

**Published:** 2025-03-20

**Authors:** Nobuhiro Kobayashi, Saori Suzuki, Yuki Sakamoto, Rigel Suzuki, Kento Mori, Yume Kosugi, Tomoya Saito, Yuan Ma, Lihan Liang, Takuma Izumi, Kisho Noda, Daisuke Okuzaki, Yumi Kanegae, Sanae Hayashi, Yasuhito Tanaka, Atsuya Yamashita, Kohji Moriishi, Yoshiharu Matsuura, Osamu Takeuchi, Tomokazu Tamura, Akinobu Taketomi, Takasuke Fukuhara

**Affiliations:** 1 Department of Gastroenterological Surgery 1, Graduate School of Medicine, Hokkaido University, Hokkaido, Japan; 2 Department of Microbiology and Immunology, Graduate School of Medicine, Hokkaido University, Hokkaido, Japan; 3 Institute for Vaccine Research and Development: HU-IVRD, Hokkaido University, Hokkaido, Japan; 4 Department of Surgery, Matsuyama Red Cross Hospital, Ehime, Japan; 5 Tokyo Bay Urayasu Ichikawa Medical Center, Chiba, Japan; 6 Genome Information Research Center, Research Institute for Microbial Diseases, Osaka University, Osaka, Japan; 7 Core Research Facilities, Research Center for Medical Sciences, The Jikei University School of Medicine, Tokyo, Japan; 8 Department of Gastroenterology and Hepatology, Faculty of Life Sciences, Kumamoto University, Kumamoto, Japan; 9 Department of Microbiology, Faculty of Medicine, Graduate Faculty of Interdisciplinary Research, University of Yamanashi, Yamanashi, Japan; 10 Division of Hepatitis Virology, Institute for Genetic Medicine, Hokkaido University, Hokkaido, Japan; 11 Center for Life Science Research, University of Yamanashi, Yamanashi, Japan; 12 Department of Molecular Virology, Research Institute for Microbial Diseases, Osaka University, Osaka, Japan; 13 Laboratory of Virus Control, Center for Infectious Disease Education and Research, Osaka University, Osaka, Japan; 14 Department of Medical Chemistry, Graduate School of Medicine, Kyoto University, Kyoto, Japan; 15 One Health Research Center, Hokkaido University, Hokkaido, Japan; 16 AMED-CREST, Japan Agency for Medical Research and Development (AMED), Tokyo, Japan; 17 Department of Virology, Faculty of Medical Sciences, Kyushu University, Fukuoka, Japan

**Keywords:** antiviral, hepatitis B virus, NEDD4-binding protein 1 (N4BP1), pregenomic RNA, RNA-binding protein

## Abstract

Chronic infection with hepatitis B virus (HBV) (chronic HBV infection) places patients at increased risk for liver cirrhosis and hepatocellular carcinoma. Although nucleos(t)ide analogues are mainly used for the treatment of HBV, they require long-term administration and may lead to the emergence of drug-resistant mutants. Therefore, to identify targets for the development of novel anti-HBV drugs, we screened for HBV-suppressive host factors using a plasmid expression library of RNA-binding proteins (RBPs). We tested the effect of 132 RBPs on HBV replication by ectopically expressing these proteins along with HBV in hepatocellular carcinoma and evaluated the intracellular capsid-associated HBV DNA level. Our screen identified NEDD4-binding protein 1 (N4BP1) as having an anti-HBV effect. In hepatocellular carcinoma cell lines transfected or infected with HBV, the overexpression of N4BP1 decreased core-associated HBV DNA levels, while knockdown or knockout of the gene encoding N4BP1 rescued core-associated HBV DNA levels. N4BP1 possesses the KH-like and RNase domains, and both were required for the anti-HBV effect of N4BP1. Additionally, we measured levels of HBV pregenomic RNA (pgRNA) and covalently closed circular DNA in the RBP-transfected cells and confirmed that N4BP1 binds pgRNA directly and regulates both the 3.5 and 2.4/2.1 kb HBV RNA. In summary, N4BP1 is a newly identified host factor able to counteract HBV production by regulating 3.5 and 2.1/2.4 kb HBV RNA.

## Data Summary

The GEO data of the RNA-seq that support the findings of this study are openly available at https://www.ncbi.nlm.nih.gov/geo/query/acc.cgi?acc=GSE225322. The accession number is GSE225322. The scRNA-seq data referred to in this study have been uploaded to the SRA under BioProject PRJNA1096305. Within the BioProject data, we referred to the following accessions: SRR28562212, SRR28562213, SRR28562215, SRR28562246, SRR28562250, SRR28562251, SRR28562253, SRR28562255, SRR28562256 and SRR28562259.

## Importance

There is still a large number of HBV-infected people in the world today because of no curative treatment for HBV infection. In this study, we focused on and screened RNA-binding proteins to identify new host factors, which inhibit HBV replication. As a result, we found that NEDD4-binding protein 1 (N4BP1) expression regulated pregenomic RNA (pgRNA), 2.4 and 2.1 kb HBV RNA by binding pgRNA. Furthermore, the KH-like domain or RNase domain of N4BP1 was involved in this anti-HBV effect. This novel factor can potentially become a key to new HBV treatments.

## Introduction

Hepatitis B virus (HBV) infection causes either acute or chronic hepatitis, with ~296 million people worldwide suffering from chronic hepatitis B and an estimated 1.5 million new cases arising each year. Chronically infected individuals are at increased risk for liver disease, and annually, ~820,000 die from cirrhosis and hepatocellular carcinoma (HCC) caused by HBV infection [1]. Although there is a prophylactic vaccine for HBV, there are still many new cases of HBV infection due to the large number of chronic HBV-infected patients still in Africa, Asia and Eastern Europe and the low vaccination rates in these highly endemic areas [2].

HBV is an enveloped DNA virus with a small, partially double-stranded genome of 3.2 kbp. Entry into hepatocytes is mediated by binding to the bile acid transporter sodium-taurocholate cotransporting polypeptide (NTCP). After being released into the nucleus, the HBV genome is converted from its relaxed circular DNA (rcDNA) form to covalently closed circular DNA (cccDNA) using the host DNA repair system [3,6]. The cccDNA serves as the transcription template for viral mRNAs, including the HBV pregenomic RNA (pgRNA). pgRNA is used to synthesize the negative-strand DNA first, and then, the positive strand is synthesized to generate rcDNA.

IFN and nucleos(t)ide analogues (NAs) are currently used as treatments for HBV infection, and both have shown efficacy in controlling viraemia [7,8]. IFNs are involved in the inhibition of HBV replication, transcription and other important processes by inducing the expression of various IFN-stimulated genes (ISGs) in host cells [9]. However, IFN treatment can cause considerable side effects, including liver damage, and is thus unsuitable for patients with severe liver dysfunction [10].

In contrast, NAs impair further HBV intrahepatic spreading by blocking the production of new virions. But the HBV HBsAg or HBeAg are not eliminated with treatment as NAs inhibit the reverse transcription step that occurs after these antigens have already been translated. Furthermore, NAs do not directly target cccDNA, allowing the virus to persist in hepatocytes [11,12]. Consequently, to prevent relapse due to reactivation of HBV after discontinuing NAs, long-term treatment is recommended [13].

In efforts to overcome the limitations of these current therapeutic options, research has looked to identify host factors with an antiviral role. Understanding the mechanism of action of such factors could aid in the development of new antiviral treatments. Several host factors are known to inhibit HBV replication, including RNA-binding proteins (RBPs). For example, the CCCH zinc finger antiviral protein ZAP (ZC3HAV1) binds to HBV RNA with its four zinc finger motifs at the N-terminus, promoting pgRNA degradation [14]. Similarly, MCPIP1 (ZC3H12A, regnase-1) has also been reported to inhibit HBV replication by binding to HBV pgRNA through its zinc finger motif, directly degrading pgRNA via its RNase activity [15]. Furthermore, PUF60 is a host factor that not only positively regulates HBV 3.5 kb precore plus pgRNA expression by binding to the HBV enhancer region II with the transcription factor TCF7L2 but can also promote degradation of the HBV 3.5 kb RNA by binding via its N-terminal RNA recognition motif [16]. Despite these descriptions, HBV produces high levels of HBV RNAs in infected primary human hepatocytes (PHHs) and i*n vivo*, so the antiviral effects of these host factors appear limited.

To determine if other RBPs may have an antiviral role, we performed a comprehensive screen using an expression library of RBPs. We identified NEDD4-binding protein 1 (N4BP1) as a host factor with anti-HBV activity. We subsequently elucidated the mechanism of how N4BP1 suppresses HBV replication by regulating HBV RNAs.

## Methods

### Cell culture

All cells were grown at 37 °C in a 5% (v/v) CO_2_ environment. Huh7 cells and HEK293T cells were obtained from the Japanese Collection of Research Bioresources Cell Bank (JCRB, Osaka, Japan, JCRB0403 and JCRB9068). HepG2-hNTCP C4 cells and HepAD38.7 cells were kindly provided by Takaji Wakita (NIID, Tokyo, Japan). PHHs were obtained from Phoenix Bio (Hiroshima, Japan). Huh7 cells and HEK293T cells were maintained in Dulbecco's Modified Eagle Medium (DMEM, Nacalai Tesque, Kyoto, Japan) supplemented with 100 U ml^−1^ of penicillin, 100 µg ml^−1^ of streptomycin (Nacalai Tesque) and 10% FBS (Sigma, MO, USA). HepG2-hNTCP C4 cells were maintained in the aforementioned media containing 400 µg ml^−1^ G418 (Nacalai Tesque). HepAD38.7 cells were maintained in DMEM/Ham’s F-12 media (Nacalai Tesque) supplemented with 10% FBS, 100 U ml^−1^ of penicillin, 100 µg ml^−1^ of streptomycin, 400 µg ml^−1^ of G418, 5 µg ml^−1^ of insulin (Nacalai Tesque) and 400 ng ml^−1^ of tetracycline (Nacalai Tesque). PHHs were maintained in PHH-specific media provided by the manufacturer (Phoenix Bio).

### Generation of RBP-overexpressing cells

RBP-overexpressing Huh7 cells were generated by transfection of RBP expression plasmids using TransIT LT-1 transfection reagent (Mirus, WI, USA). Briefly, Huh7 cells were seeded in 24-well plates (Greiner, Bad Haller, Austria) 1 day prior to the transfection. The following day, 1 µl of TransIT LT-1 transfection reagent, 0.3 µg of RBP expression plasmid and 0.1 µg of a plasmid containing a 1.3-fold-overlength genome of HBV (genotype C) were mixed in 50 µl of Opti-MEM (Thermo Fisher Scientific, MA, USA). After 15-min incubation at room temperature, the mixture was added to the supernatant of the cultured Huh7 cells. Entecavir (ETV) (Fujifilm, Tokyo, Japan) was added at 5 µM the day after transfection as a positive control.

RBP-overexpressing HepG2-hNTCP C4 cells were made by lentiviral transduction. Lentiviruses were made by transfecting HIV gag/pol, VSV-G, REV and pcs-II EF RBP expression plasmids into HEK293T cells using TransIT LT-1 transfection reagent. At 2 days post-transfection (d.p.t.), the supernatant of HEK293T was collected and spun at 4,200 r.p.m. for 2 min at room temperature, and the supernatant was added to HepG2-hNTCP C4 cells. ETV or heparin was used as a positive control. ETV was added at 5 µM the day after infection. Heparin (Mochida Pharmaceutical Co., Tokyo, Japan) was added at 5 U ml^−1^ 30 min before infection.

To express N4BP1 in PHHs, we used an adenovirus vector (AdV). The N4BP1-expressing AdV (N4BP1 AdV) was constructed using the cosmid cassette pAxEFwit2, which has a cloning site *SwaI* flanked by the EF1*α* promoter and rabbit *β*-globin polyA signal in the E1-deleted region of the adenovirus genome. The N4BP1 cDNA was prepared from the pCS2-EF-N4BP1-HA plasmid and inserted into the *SwaI* cloning site. The AdV was prepared as previously described [17,18]. The AdV was titrated using the methods described by Pei *et al*. [19]. Briefly, the copy numbers of the viral genome successfully transduced into target cells were measured by quantitative PCR (qPCR). For generating N4BP1-overexpressing PHHs, cells were infected with N4BP1 AdV at an m.o.i. of 5 or 30 2 days before HBV infection.

### Generation of RBP knockdown cells

RBP knockdown cell lines were generated using Silencer Select siRNA (Thermo Fisher Scientific) and Lipofectamine RNAiMAX (Thermo Fisher Scientific) following the manufacturer’s protocol. Briefly, 1.5 µl of Lipofectamine RNAiMAX and 5 pg of siRNA were mixed in 50 µl of Opti-MEM. After incubating for 5 min at room temperature, the mixture was gently added to the cell supernatant. The following siRNA reagents were used in this study: control siRNA (Silencer Select Negative Control No. 1 siRNA, 4390843), ZCCHC10 siRNA#1 (s29507), ZCCHC10 siRNA#2 (s29508), ZCCHC10 siRNA#3 (s29509), HNRNPC siRNA#1 (s6719), HNRNPC siRNA#2 (s6720), HNRNPC siRNA#3 (s6721), KHNYN siRNA#1 (s23623), KHNYN siRNA#2 (s23624), N4BP1 siRNA#1 (s18638) and N4BP1 siRNA#2 (s18639).

For PHH, 0.5 µl of Lipofectamine RNAiMAX and 5 nM of siRNA were mixed in 50 µl of Opti-MEM 2 days before HBV (genotype D) infection. Infection to PHH was performed at 100 genome equivalents (GEq)/cell in PHH-specific media containing 4% polyethylene glycol (PEG) 8000 following the manufacturer’s protocol. The culture media were replaced every 4 days. Cells were harvested at 7 and 10 days post-infection (d.p.i.) for reverse transcription qPCR (RT-qPCR).

### Generation of N4BP1 knockout cells

N4BP1 knockout (KO) Huh7 cells were generated by CRISPR/Cas9 using pX330-U6-Chimeric_BB-CBh-hSpCas9 (pX330, Addgene, MA, USA) and HR110PA-1 (System Biosciences, CA, USA). The pX330-puro vector was constructed by integrating the puromycin resistance gene sequence from the HR110PA-1 vector into the pX330 vector. The pX330-puro vector was digested using *BbsI* (Biolabs, MA, USA), and the target sequence was subcloned. The plasmid was transfected into Huh7 cells using TransIT LT-1 transfection reagent, and after selection with puromycin dihydrochloride (InvivoGen, CA, USA), a single colony was isolated using cloning cylinders (Merck, Darmstadt, Germany) and propagated. KO of the target gene was confirmed by western blotting (WB) and DNA sequencing.

### Plasmid constructions

We used the following plasmids containing 1.3-fold-overlength genomes of different HBV genotypes: pUC19-Ae_us (genotype A, accession no. AB246337), pGEM-Bj-JPN56 (genotype B, accession no. AB246342) and pUC19-C_JPNAT (genotype C, accession no. AB246345).

Each RBP expression plasmid had a C-terminus HA tag and was inserted between the *XhoI* and *XbaI* sites of the pcs-II EF vector. The KH-like domain deletion mutant (ΔKH), RNase domain deletion mutant (ΔRNase) and D623N mutant were generated from the N4BP1 expression plasmid. ΔKH plasmid was generated by deleting aa 59–143 from N4BP1; ΔRNase was generated by deleting aa 617–769 N4BP1; and D623N was generated by changing the 623rd aa from asparagine acid to asparagine. To insert these mutated N4BP1 genes into the pcs-II EF vector between the *XhoI* and *XbaI* sites, we used the following primer sets and In-Fusion Snap Assembly Master Mix (Takara Bio, Shiga, Japan) per the manufacturer’s directions. Each N4BP1 mutant plasmid had a C-terminus HA tag.

ΔKH: 5′-CGCTACCGGTCTCGAGATGGCGGCCCGGGCGGTGCT-3′ (forward)

5′-GGGTAGGTTCTCTTTCGCCCCGCAGAGCTGCAGCC-3′ (reverse), and

5′-CAGCTCTGCGGGGCGAAAGAGAACCTACCCAGTAG-3′ (forward)

5′-GGTACATGGTCTCGAGATCCAACACCATGGCAGAAA-3′ (reverse)

ΔRNase: 5′-CGCTACCGGTCTCGAGATGGCGGCCCGGGCGGTGCT-3′ (forward)

5′-TTCCTTCTGAAGAAAATCCGTTCTCCCTGGTTCAT-3′ (reverse), and

5′-CCAGGGAGAACGGATTTTCTTCAGAAGGAAGTCTG-3′ (forward)

5′-GGGTAGGTTCTCTTTCGCCCCGCAGAGCTGCAGCC-3′ (reverse)

D623N: 5′-CGCTACCGGTCTCGAGATGGCGGCCCGGGCGGTGCT-3′ (forward)

5′-AACATTGCTCCCATTTATAACAATGTGTTTCAAAT-3′ (reverse), and

5′-AATGGGAGCAATGTTGCAATTACCCATGGTCTGAA-3′ (forward)

5′-GGTACATGGTCTCGAGATCCAACACCATGGCAGAAA-3′ (reverse)

### Viruses

The production of HBV particles (genotype D) was adapted from a previous report [20]. Briefly, HepAD38.7 cells were cultured under tetracycline (400 ng ml^−1^) for maintenance. To produce HBV particles, tetracycline was removed and the supernatant was collected after 6 and 9 days. The collected supernatant was mixed with 30% PEG 8000 (Sigma) and incubated overnight at 4 °C. Then, it was spun at 3,000 r.p.m. for 20 min at 4 °C for concentration. Before HBV infection, HepG2-hNTCP C4 cells were seeded in collagen type I-coated 24-well plates (Iwaki, Tokyo, Japan) and transduced with lentivirus to express RBP (see ‘Generation of RBP-overexpressing cells’). At 2 days post-transduction, the cells were infected with 5,000 GEq/cell of HBV in DMEM containing 3% DMSO (Nacalai Tesque) and 4% PEG 8000, and the medium was changed every 2–3 days until 14 d.p.i.

PHH was infected with HBV genotype C purchased from Phoenix Bio or HBV genotype D generated as described above. The infection into PHHs was performed at 5 GEq/cell (genotype C) or 100 GEq/cell (genotype D) in PHH-specific media containing 4% PEG 8000 following the manufacturer’s protocol. The culture media were replaced every 4 days. Cells were harvested at 7, 10 and 14 d.p.i. for WB and RT-qPCR.

### Western blotting

Cells were lysed with 2× Laemmli sample buffer (Bio-Rad Laboratories, CA, USA) containing 10% 2-mercaptoethanol (Sigma) and boiled for 10 min at 100 °C. Then, lysates were loaded on a 10–20% gradient SDS-PAGE gel (Atto, Tokyo, Japan) and transferred onto a polyvinylidene fluoride transfer membrane (Millipore, MA, USA). Membranes were immersed in Block Ace (Kac, Kyoto, Japan) containing 1% BSA (Sigma) and incubated for 1 h at room temperature. The blocking solution was then decanted and replaced with Can Get Signal Solution 1 (Toyobo, Osaka, Japan) containing the appropriate antibodies described below and incubated for 1 h. The membranes were washed with 1× PBS (Fujifilm) containing 0.1% polyoxyethylene [21] sorbitan monolaurate (Fujifilm) and incubated with Can Get Signal Solution 2 (Toyobo) containing horseradish peroxidase-conjugated antibody against rabbit or mouse immunoglobulins for 1 h. After the addition of Amersham ECL Prime WB Detection Reagents (Cytiva, Tokyo, Japan), the membranes were subsequently imaged via LuminoGraph I (Atto).

The following antibodies were used in this study: anti-N4BP1 (Bethyl Laboratories, TX, USA), anti-ZC3H12B (Gene Tex, CA, USA), anti-HNRNPC (abcam, Cambridgeshire, UK), anti-ZCCHC10 (Sigma), anti-KHNYN (Fujifilm), anti-HA tag (Biolegend, CA, USA), anti-rabbit IgG (H+L) HRP (Jackson Immuno Research Laboratories, MD, USA), anti-mouse IgG (H+L) HRP (Thermo Fisher Scientific) and anti-GAPDH (Fujifilm). The antibodies used in this study are listed in Table S2 (available in the online Supplementary Material).

### Purification of intracellular core-associated HBV DNA

To extract intracellular HBV DNA, cells were washed twice with PBS, and cells were lysed in lysis buffer [100 mM Tris-HCl (pH 8.0), 0.2 % NP-40] for 15 min at 4 °C. After spinning at 13,000 r.p.m. for 1 min at room temperature, the supernatant was collected and incubated with 1 M MgCl_2_, 0.2 mg ml^−1^ of DNase I (Sigma) and 10 mg ml^−1^ of RNase A (Nacalai Tesque) for at least 3 h at 37 °C. After adding 500 mM EDTA and 5 M NaCl, the lysates were digested with 20 mg ml^−1^ of proteinase K (Kanto Chemical, Tokyo, Japan) and 10% SDS for 5 h at 55 °C. The extracted HBV DNA was purified using phenol-chloroform-isoamyl alcohol (Sigma) and then precipitated with isopropanol, 0.5 mg ml^−1^ of glycogen and 3 M sodium acetate. After washing with 70% ethanol, the purified HBV DNA was resolved in 50 µl pure water and detected by qPCR using Power SYBR Green PCR Master Mix (Applied Biosystems, CA, USA). As a standard for core-associated HBV DNA, pUC19-C_JPNAT was used. qPCR procedures were following the manufacturer’s protocol. Briefly, the samples were incubated at 50 °C for 2 min and at 95 °C for 10 min for initial denaturation. Then, the samples were incubated at 95 °C for 15 s and at 60 °C for 1 min for 40 cycles of polymerase reaction. The following primer pair was used in this study.

core-associated HBV DNA: 5′-GGAGGGATACATAGAGGTTCCTTGA-3′ (forward)

5′-GTTGCCCGTTTGTCCTCTAATTC-3′ (reverse)

### Quantification of intracellular HBV cccDNA level

To extract intracellular HBV cccDNA, the cells were lysed by lysis buffer [10 mM Tris-HCl (pH 7.4), 10 mM EDTA, 0.5% SDS] and 5 M NaCl was added. After incubation overnight at 4 °C, the samples were spun at 15,000 r.p.m. for 30 min at 4 °C, and the supernatant was collected to isolate protein-free DNA. The DNA was purified a total of three times by phenol (Nacalai Tesque), phenol-chloroform-isoamyl and chloroform (Fujifilm). After precipitation with isopropanol and ethanol, Plasmid-Safe ATP-Dependent DNase (Lucigen, WI, USA) was added to the purified DNA to enrich HBV cccDNA through degrading the unwanted linear HBV DNA forms. The purified DNA was extracted in 30 µl of 10 mM Tris-Cl (pH 8.5) using the FastGene Gel/PCR Extraction Kit (NIPPON Genetics, Tokyo, Japan). HBV cccDNA was measured by qPCR using TaqMan Fast Advanced Master Mix (Applied Biosystems). As a standard for cccDNA, pUC19-C_JPNAT was used. qPCR procedures were done per the manufacturer’s protocol. In brief, the samples were incubated at 50 °C for 2 min and at 95 °C for 10 min to initiate denaturation. Then, the samples were incubated at 95 °C for 15 s and at 60 °C for 1 min for 50 cycles for the polymerase reaction. The following primer pair and probe were used in this study [20].

cccDNA: 5′-CGTCTGTGCCTTCTCATCTGC-3′ (forward)

5′-GCACAGCTTGGAGGCTTGAA-3′ (reverse)

5′-CTGTAGGCATAAATTGGT-3′ (probe)

### Measurement of intracellular HBV pgRNA

Total RNA including pgRNA was extracted using the RNeasy Mini Kit (Qiagen, Hilden, Germany) following the manufacturer’s protocol. The extracted RNA was resolved in 50 µl pure water and measured by RT-qPCR using Power SYBR Green RNA-to-Ct 1-step Kit (Applied Biosystems). The pgRNA expression level was normalized against the expression level of GAPDH as a housekeeping gene and calculated by the ΔΔCT method. RT-qPCR procedures were done following the manufacturer’s protocol. First, the samples were incubated at 50 °C for 2 min and at 95 °C for 10 min to initiate denaturation. Then, the samples were incubated at 95 °C for 15 s and at 60 °C for 1 min for 40 cycles for the polymerase reaction. Samples were then incubated at 95 °C for 15 s, 60 °C for 1 min and 95 °C for 15 s for the melting curve measurement. The following primer pairs were used in this study.

pgRNA: 5′-TCCCTCGCCTCGCAGACG-3′ (forward)

5′-GTTTCCCACCTTATGAGTC-3′ (reverse)

GAPDH: 5′-AGAAGGCTGGGGCTCATTTG-3′ (forward)

5′-AGGGGCCATCCACAGTCTTC-3′ (reverse)

### Immunostaining

Huh7 cells and HepG2-hNTCP C4 cells were seeded on a normal chamber slide (Corning, NY, USA) or collagen-coated chamber slide (Iwaki), respectively. On the next day, cells were washed by PBS twice and fixed with 4% paraformaldehyde (Nacalai Tesque) in PBS for 15 min and permeabilized with 0.2% Triton X-100 (Nacalai Tesque) in PBS for 20 min. After blocking with 2% BSA in PBS for 1 h, anti-N4BP1 antibody (Bethyl) in 2% BSA (Sigma) in PBS was reacted for overnight. After washing with PBS three times, the secondary antibody (Alexa-488, Thermo Fisher Scientific) was reacted for 1 h. After washing with PBS three times, the cover glass was placed on the chamber slide with Vibrance Antifade Mounting Medium with DAPI (Vector Laboratories, Inc., Newark, USA). All images were acquired with Nikon A1R HD25 microscope (Nikon, Tokyo, Japan).

### pgRNA degradation assay

HepAD38.7 cells were seeded in collagen type I-coated 12-well plates (Iwaki) and cultured overnight in media containing tetracycline hydrochloride. After washing twice with PBS, cells were cultured without tetracycline for 48 h. Then, the cells were transfected with N4BP1 WT expression plasmid, ΔKH expression plasmid, ΔRNase expression plasmid or control vector. These plasmids were transfected as follows: 100 µl of Opti-MEM, 6 µl of TransIT LT-1 and 3 µg of expression plasmid. After transfection of plasmids, cells were cultured for 48 h and changed to a tetracycline-containing media in order to stop HBV production. For core-associated HBV DNA, cells were collected for 5 days prior to tetracycline addition. For pgRNA, cells were collected at 2, 3 and 4 days after changing to tetracycline-containing media. Core-associated HBV DNA and total RNA, including pgRNA, were extracted as described above for RT-qPCR.

### Drug treatment assay

Huh7 cells, HepG2-hNTCP C4 cells and PHHs were stimulated with 1,000 U ml^−1^ of IFN-*α* (PBL Assay Science, NJ, USA) or 100 ng ml^−1^ of IFN-*λ* (PeproTech, NJ, USA). At 6, 24 and 48 h post-drug stimulation, total RNA in cells was extracted for qPCR using the RNeasy Mini Kit, and cDNA was synthesized using SuperScript IV VILO Master Mix (Invitrogen). For WB, the cells were lysed with 2× Laemmli sample buffer containing 10% 2-mercaptoethanol and processed as described above. qPCR was performed using Power SYBR Green PCR Master Mix. The mRNA expression levels of N4BP1 and ISG15 were normalized against the expression level of GAPDH as a housekeeping gene and calculated by the ΔΔCT method. The following primer pairs were used in this study.

N4BP1 : 5′-CCCGATGATCCTCTGGGAAG-3′ (forward)

5′-TTTGGCAGGGCACTGAGTAG-3′ (reverse)

ISG15 : 5′-ACTCATCTTTGCCAGTACAGGAG-3′ (forward)

5′-CAGCATCTTCACCGTCAGGTC-3′ (reverse)

GAPDH: see above

### Northern blotting

Huh7 cells were co-transfected with a plasmid containing the 1.3-fold-overlength genome of HBV genotype C and N4BP1 using TransIT LT-1 transfection reagent, and cells were collected at 3 d.p.t. Total RNA was extracted using Isogen (Nippon Gene, Tokyo, Japan) and subjected to electrophoresis in 1% agarose-formaldehyde gel and morpholinepropanesulfonic acid buffer (Nacalai Tesque). rRNA was visualized by ethidium bromide staining, and electrophoresed RNA was transferred onto a positively charged nylon membrane (Roche, Basel-Stadt, Switzerland). An HBV RNA probe and GAPDH RNA probe were linearized and synthesized by digoxigenin RNA labelling kit (Roche). Hybridization and detection were performed using the DIG Northern Starter kit (Roche) following the manufacturer’s protocol. The signals were detected by LuminoGraph I (Atto).

### RNA immunoprecipitation assay

Huh7 cells were co-transfected with a plasmid containing the 1.3-fold-overlength genome of HBV genotype C and HA-tagged N4BP1 WT or domain-deficient mutants or HA control vector using TransIT LT-1 transfection reagent, and cells were collected at 3 d.p.t. Cells were collected and immunoprecipitated with anti-HA-tag monoclonal antibody magnetic beads (Medical and Biological Laboratories Co., Ltd., Tokyo, Japan). Immunoprecipitation (IP) and RNA extraction were performed using RiboCluster Profiler RIP-Assay Kit (Medical and Biological Laboratories Co., Ltd.) following the manufacturer’s protocol. WB and pgRNA qPCR were measured as described above.

### HBeAg Chemiluminescence Immunoassay and HBsAg ELISA

N4BP1-overexpressing PHHs were infected with HBV genotype C (5 GEq/cell). At 14 d.p.i., RNA was extracted from the cells and pgRNA was measured as described above. HBV proteins in the supernatant were assessed as follows: HBeAg Chemiluminescence Immunoassay (CLIA, Ig Biotechnology, Burlingame, USA) and HBs S Antigen Quantitative ELISA Kit, Rapid-II (Takara Bio) following manufacturers’ protocol.

### HBV promoter assay

The Huh7 GL4.18 CURS_BCP_AeUS, Huh7 GL4.18 EnhIX-Pro_AeUS, Huh7 GL4.18 preS1-Pro_AeUS and Huh7 GL4.18 preS2-Pro_AeUS cell lines were established as described previously [22]. These HBV promoter reporter cell lines were transfected with either pcs-II EF N4BP1 or pcs-II EF GFP plasmid, together with the pGL4.77 *β*-actin plasmid, using the TransIT-LT1 transfection reagent (Mirus). The pGL4.77 *β*-actin plasmid was constructed by inserting the *β*-actin promoter fragment (Xba I/Hind III) from pDRIVE5SEAP-hβAct/RU5’ (InvivoGen) into the Nhe I/Hind III sites of pGL4.77 (hRlucP/Hygro) (Promega). The pGL4.77 *β*-actin plasmid was used to normalize transfection efficiency. Luciferase activity in the resulting cells was measured using the Dual-Luciferase Reporter Assay System (Promega).

### RNA sequencing

PHHs were analysed for RNA sequencing. N4BP1-overexpressing PHHs were infected with HBV genotype C (5 GEq/cell) as described above. At 3 d.p.i., total RNA in PHHs was extracted using RNeasy Mini Kit following the manufacturer’s protocol. RNA sequencing was performed by Osaka University (Genome Information Research Center, Research Institute for Microbial Diseases, Osaka University, Osaka, Japan). We ran two-well (N4BP1-overexpressing) vs two-well (control) samples. Whole transcriptome sequencing was applied to RNA samples using the Illumina NovaSeq 6000 platform in a 101 bp single-end mode. Sequenced reads were mapped to the human reference genome sequence (hg19) using TopHat version 2.2.1 in combination with Bowtie 2 version 2.2.3 and SAMtools version 1.0. The number of fragments per kb of exon per million mapped fragments was calculated using Cufflinks version 2.2.1. Following the exclusion of genes with expression counts below 50, differential expression analysis was conducted utilizing the DESeq2 package (version 1.42.0) [23]. Genes were considered significantly differentially expressed if they satisfied the criteria of an adjusted *P*-value less than 0.1 and an absolute fold change greater than 2. Subsequently, pathway enrichment analysis of the differentially expressed genes was performed using the Kyoto Encyclopedia of Genes and Genomes database [24] with the clusterProfiler package (version 4.10.0) [25]. Plots were generated using the ggplot2 package (version 3.5.1) [26]. To review GEO data, access https://www.ncbi.nlm.nih.gov/geo/query/acc.cgi?acc=GSE225322. The accession no. is GSE225322.

### Analysis of N4BP1 expression in clinical liver samples

To assess the N4BP1 expression of HBV-HCC resection samples, we confirmed that all specimens were HBs-Ag (+), HBe-Ag (−) and HBe-Ab (+) as documented in the patient medical records. Based on HBV DNA data in the serum, the samples were divided into an HBV DNA-positive group (*n*=9) and an HBV DNA-negative group (*n*=8). In all cases, RNA was extracted from the non-cancerous areas of resected tissues using the RNeasy Mini Kit, and *N4BP1* and *ISG15* mRNA expressions were measured by qPCR. This study was approved by the Institutional Review Board of the Graduate School of Medicine, Hokkaido University (no. 14-015, 022–-0195). All patients participating in this study provided written informed consent for the use of their samples and clinical data, and they were informed of the opportunity to opt out.

### Analysis of scRNA-seq

To assess N4BP1 expression of patients with chronic HBV infection (CHB), publicly available scRNA-seq data obtained from liver biopsies [27] were analysed. Patients in phases 3 and 4 of CHB classified by the European Association for the Study of the Liver [28] were compared with patients who were functionally cured. Phases 3 and 4 were categorized as inactive carrier and HBeAg-negative chronic hepatitis, respectively. Phases 3 and 4 are defined as HBeAg negative, HBsAg low to intermediate and low HBV DNA. All public data were downloaded using sratoolkit version 3.0.0. The data were processed using the cellranger version 8.0.1 pipeline [29] and mapped to the GRCh38 genome, and annotation data were constructed by 10× Chromium. The resulting raw feature-barcode matrix was then imported into scanpy version 1.7.1 [30]. Scaling and normalization were performed using methods consistent with those in the study where the scRNA-seq data were originally obtained. Subsequently, cell annotations were conducted using Azimuth [31]. Based on the cell annotations, the average expression levels of *N4BP1* and *ISG15* were analysed in their respective positive hepatocytes.

### Statistical analysis

Data are averages of three or more independent experiments. All data in figures are presented as the mean±sd. The evaluation of data was performed by the Mann–Whitney U test. *P*-value of <0.05 was considered statistically significant. Statistical analysis was performed using JMP Pro version 16.

## Results

### Screen for RBPs that inhibit HBV propagation

**Fig. 1.**
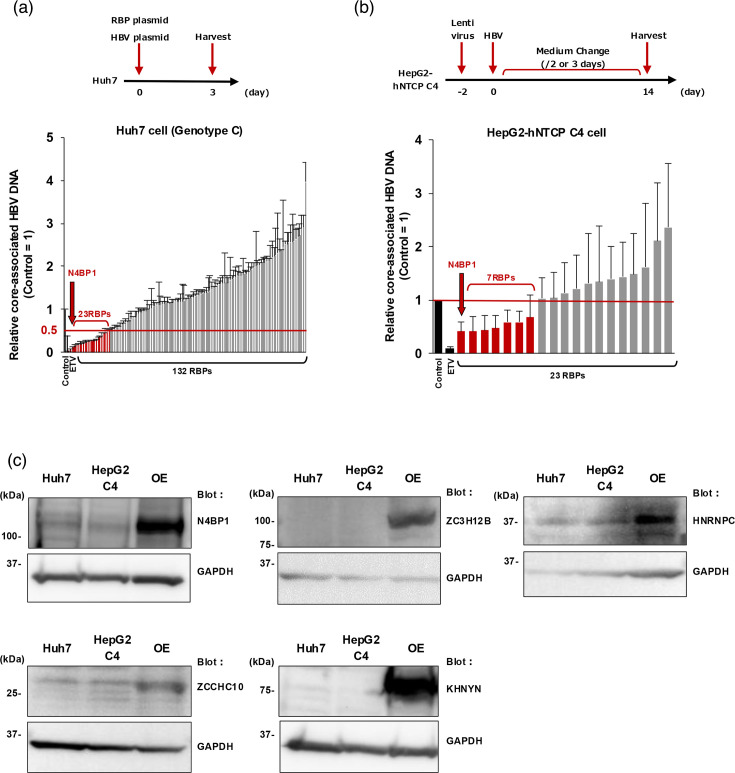
Screening for RBPs that inhibit HBV propagation. (a) A plasmid containing the 1.3-fold-overlength genome of HBV genotype C and 132 RBP expression plasmids were co-transfected into Huh7 cells. Intracellular core-associated HBV DNA levels were assessed at 3 d.p.t. Using the 50% of core-associated HBV DNA level of the control as a cutoff, 23 out of the 132 RBPs suppressed core-associated HBV DNA levels. (b) HepG2-hNTCP C4 cells overexpressing the 23 RBPs were infected with HBV genotype D. At 14 d.p.i., cells were harvested and intracellular core-associated HBV DNA levels were measured by qPCR. Seven out of the 23 RBPs (N4BP1, TIA-1, ZC3H12B, PJA1, HNRNPC, ZCCHC10 and KHNYN) reduced core-associated HBV DNA levels in comparison with the control. (c) WB analyses in naïve Huh7 cells and HepG2-hNTCP C4 cells of the five factors (N4BP1, ZC3H12B, HNRNPC, ZCCHC10 and KHNYN) identified in the secondary screening. As a positive control, WB was performed on Huh7 cells transfected to overexpress each factor. Error bars represent the sd. HepG2 C4, HepG2-hNTCP C4; OE, overexpression.

To identify novel RBPs involved in HBV propagation, we screened 132 RBPs using an expression plasmid library. Huh7 cells were co-transfected with an RBP expression plasmid and a 1.3-fold-overlength HBV genome (genotype C). At 3 d.p.t., cells were collected to measure intracellular core-associated HBV DNA. As shown in Fig. 1(a), 23 out of 132 RBPs suppressed core-associated HBV DNA levels by at least 50% compared with the control.

As a secondary screen, we then examined the effect of these 23 RBPs on HBV entry and replication. HepG2-hNTCP C4 cells expressing each of the 23 RBPs were infected with HBV (genotype D), and intracellular core-associated HBV DNA was measured by qPCR at 14 d.p.i. Seven of the 23 RBPs (N4BP1, TIA-1, ZC3H12B, PJA1, HNRNPC, ZCCHC10 and KHNYN) reduced core-associated HBV DNA levels in comparison with the control (Fig. 1b). These results were consistent with previous reports that found TIA-1 and PJA1 inhibit the expression of HBsAg and HBV transcription and replication, respectively [32,33].

**Fig. 2.**
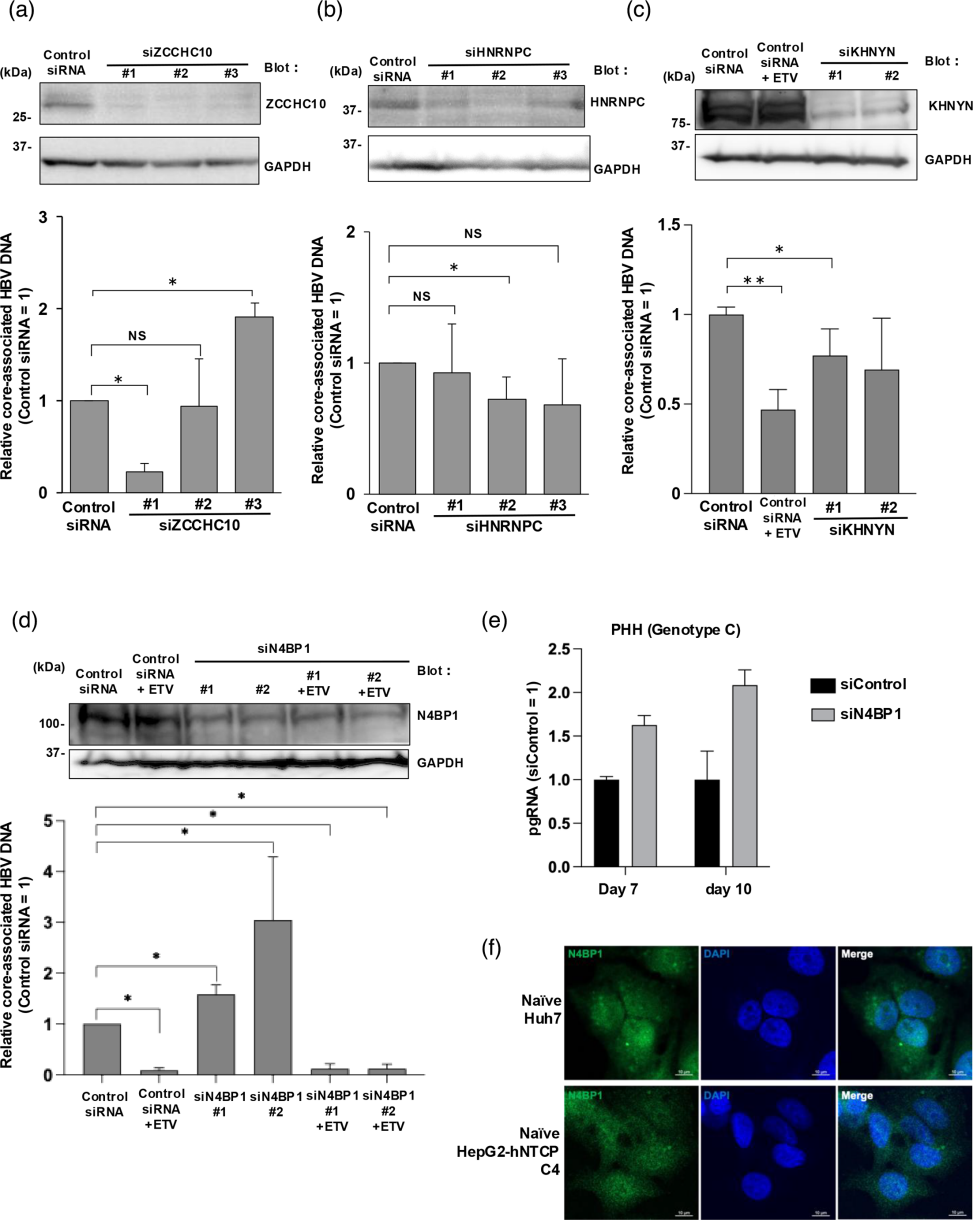
Measurement of core-associated HBV DNA levels after RBP knockdown. (a) Huh7 cells were transfected with siRNA and then co-transfected with the 1.3-fold-overlength genome of HBV genotype C and an RBP expression plasmid. WB analyses and qPCR of core-associated HBV DNA were performed at 3 d.p.t. ZCCHC10 (a), HNRNPC (b), KHNYN (c) and N4BP1 (d). GAPDH was used as a loading control and ETV was used as a positive control. (e) PHHs were transfected with N4BP1 siRNA 2 days before HBV (genotype D) infection, and cells were collected at 7 and 10 d.p.i. and pgRNA was measured. (f) Naïve Huh7 cells (upper) and naïve HepG2-hNTCP-C4 (lower) were seeded on a chamber slide with/without collagen coated. On the next day, cells were stained by anti-N4BP1 antibody and visualized with Alexa-488 secondary antibody (green) with DAPI (blue). Scale bar indicates 10 µm. Error bars represent the sd. *P<0.05, **P<0.01, as determined by the Mann–Whitney U test. All qPCR experiments were independently performed at least three times. ns, not significant.

Of the five remaining factors, we found by WB that four (N4BP1, HNRNPC, ZCCHC10 and KHNYN) are endogenously expressed in Huh7 and HepG2-hNTCP C4 cells (Fig. 1c). To test whether these proteins affect HBV replication, we knocked down each one and assessed core-associated HBV DNA levels following transfection with the 1.3-fold-overlength genome of HBV genotype C. The antiviral drug ETV, a nucleoside analogue, was used as a positive control. Knockdown of each factor was confirmed by WB, but unlike knockdown of N4BP1, knockdown of ZCCHC10, HNRNPC and KHNYN did not consistently increase the levels of core-associated HBV DNA depending on the siRNA used (Fig. 2a–d). Thus, N4BP1 became the focus of our study. Also, we confirmed that N4BP1 knockdown increased pgRNA in PHHs (Fig. 2e). N4BP1 #2 siRNA was transfected into PHHs to generate N4BP1-knockdown PHHs 2 days before HBV infection, and pgRNA levels were increased at 7 and 10 d.p.t. compared with control siRNA (Fig. 2e). The localization of N4BP1 in naïve Huh7 cells and in naïve HepG2-hNTCP C4 cells is indicated in both the cytoplasm and nucleus (Fig. 2f).

### N4BP1 is a suppressive host factor of HBV replication

**Fig. 3.**
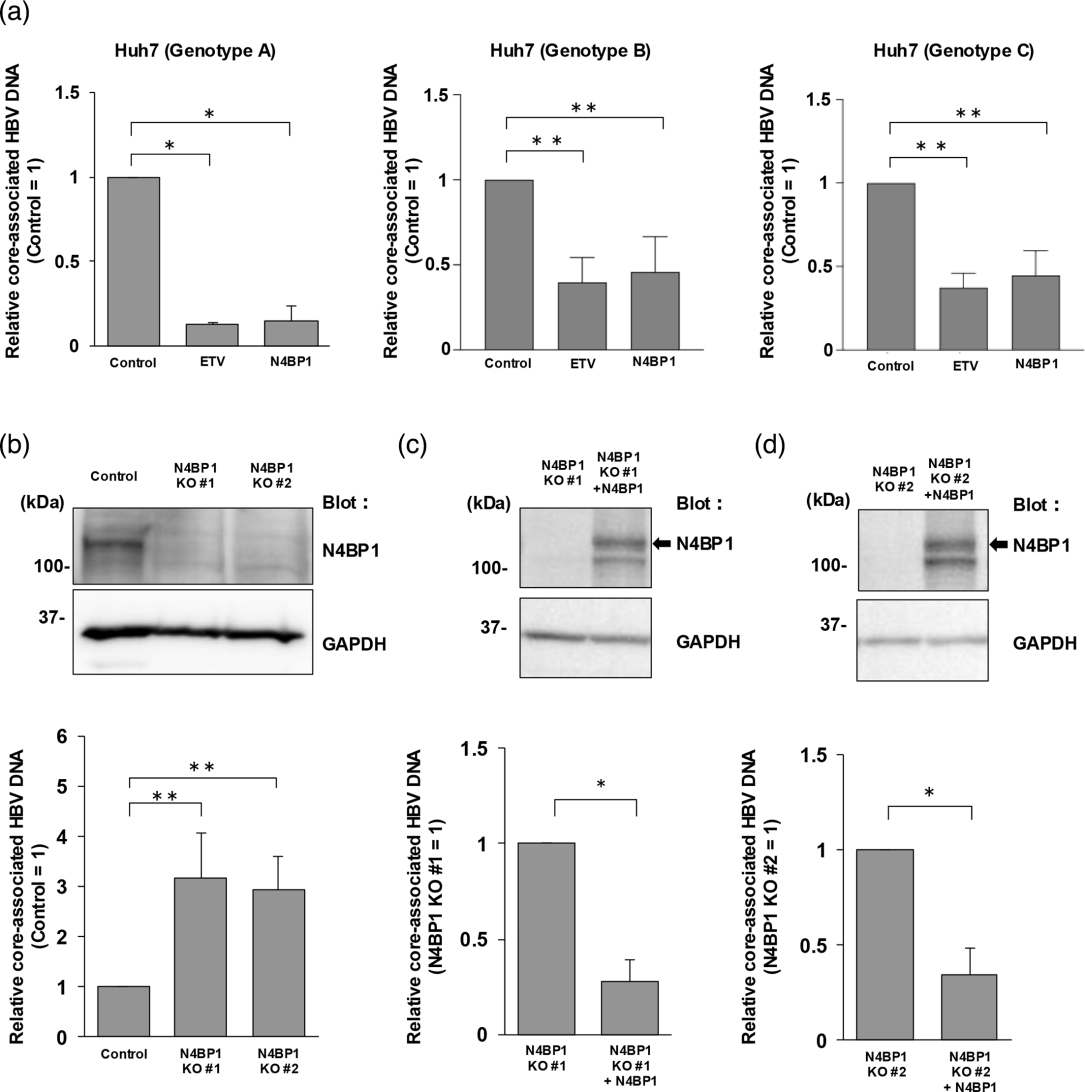
N4BP1 is a suppressive host factor of HBV replication. (a) Plasmids containing 1.3-fold-overlength genomes of HBV genotypes A, B or C were co-transfected with N4BP1 expression plasmids into Huh7 cells. At 3 d.p.t., the core-associated HBV DNA levels were measured by qPCR. Genotype A (left), genotype B (middle) and genotype C (right). (b) WB analysis and core-associated HBV DNA levels of N4BP1 KO Huh7 cells. N4BP1 KO Huh7 cells were transfected with the plasmid containing 1.3-fold-overlength genomes C, and WB and qPCR were performed at 3 d.p.t. N4BP1 expression by WB (upper, anti-N4BP1). GAPDH was used as a loading control (lower, anti-GAPDH). Core-associated HBV DNA levels were increased relative to control in both N4BP1-KO lines that were generated. N4BP1 KO Huh7 cell lines #1 (c) and #2 (d) were transfected with the N4BP1 plasmid to restore N4BP1 expression (upper, anti-N4BP1). GAPDH was used as a loading control (lower, anti-GAPDH). In the N4BP1-rescued KO Huh7 cells, the level of core-associated HBV DNA was significantly lower than in the non-rescued N4BP1 KO Huh7 cells. Error bars represent the sd. *P<0.05, **P<0.01, as determined by the Mann–Whitney U test. All qPCR experiments were independently performed at least three times.

Next, to determine whether N4BP1 also had an antiviral effect against other HBV genotypes, we co-transfected Huh7 cells with a plasmid expressing N4BP1 and a plasmid containing 1.3-fold-overlength genomes of HBV genotypes A, B and C. At 3 d.p.t., core-associated HBV DNA levels were decreased in cells transfected with each genotype (Fig. 3a).

To further elucidate the role of N4BP1 in HBV replication, we constructed Huh7 N4BP1 KO cells using CRISPR/Cas9. We generated two clones of N4BP1 KO Huh7 cells (Fig. 3b) and once more assessed whether intracellular core-associated HBV DNA levels changed at 3 d.p.t. with a plasmid containing the 1.3-fold-overlength genome of HBV genotype C. In both KO lines, core-associated HBV DNA levels increased approximately threefold compared with control cells (Fig. 3b). After rescuing N4BP1 expression in our KO line, core-associated HBV DNA levels became significantly decreased again (Fig. 3c, d), suggesting that N4BP1 is a suppressive host factor of HBV replication.

**Fig. 4.**
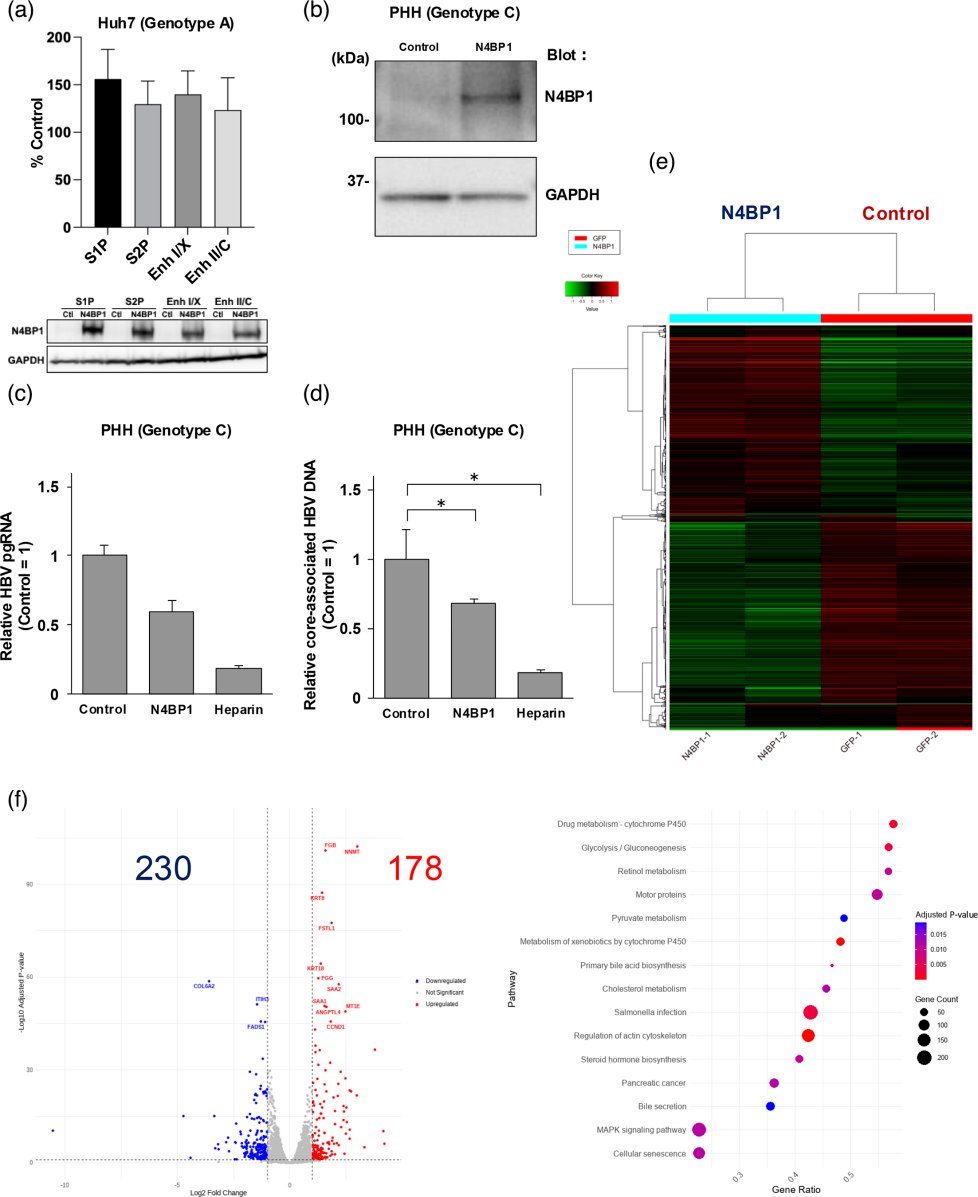
N4BP1 does not affect HBV promoters nor transcriptional factors. (a) The HBV CURS BCP, EnhI-X, PreS1 or PreS2 promoter (genotype A) stable expression cell lines were co-transfected with a plasmid with N4BP1 and *β*-actin using TransIT LT-1 transfection reagent. *β*-Actin-encoded Renilla luciferase was used to normalize transfection efficiency. The transfected cells were harvested 48 h post-transfection, and firefly and Renilla luciferase were measured (upper). WB analysis of Huh7 cells expressing N4BP1 (middle, anti-N4BP1). GAPDH was used as a loading control (lower, anti-GAPDH). PHHs were infected with AdV 2 days before HBV (genotype C) infection, and cells were collected at 14 d.p.i. (b) WB analysis of PHHs expressing N4BP1 using AdVs (upper) and GAPDH (lower). Comparison of pgRNA (c) and core-associated HBV DNA (d) levels in N4BP1-overexpressing PHHs and control at 14 d.p.i. Heparin was used as a positive control. (e) Heat map of the transcriptomic profiles of N4BP1-expressing and control PHHs infected with HBV. (f) In the volcano plot, we set a cutoff value of gene expression at absolute fold change greater than 2 and an adjusted *P*-value less than 0.1 compared with control cells. Relative to the control cells, 178 factors were upregulated, and 230 factors were downregulated in the N4BP1-overexpressing PHHs. (g) Pathway enrichment analysis of the differentially expressed genes in N4BP1-overexpressing PHHs vs the control PHHs demonstrates the distinct differences in pathways induced by N4BP1 overexpression. The graph depicts the number of genes corresponding to each pathway through the size of its dots. The colour gradient, which transitions from red to purple, illustrates the adjusted *P*-value indicating the significance of enrichment. The gene ratio on the horizontal axis indicates the proportion of input genes that are annotated to each term, thereby highlighting the distinct impact of N4BP1 overexpression. The raw data sets are shown in Table S1.

Next, we assessed whether cccDNA activity was affected by N4BP1. To determine whether N4BP1 affects cccDNA activity resulting in anti-HBV effect, Huh7 cells that express firefly luciferase under the control of the HBV CURS BCP, EnhI-X, PreS1 or PreS2 promoter (genotype A) were co-transfected with an N4BP1/control plasmid and *β*-actin plasmid encoding Renilla luciferase. *β*-Actin was used to standardize the transfection efficiency. Firefly and Renilla luciferase activities were measured at 2 d.p.t., and all the promoter activities were not reduced by N4BP1 (Fig. 4a).

In addition, we performed a transcriptomic analysis of N4BP1-overexpressing PHHs vs control PHHs to assess whether N4BP1 affects regulatory transcription factors that might affect HBV replication. First, we confirmed that N4BP1 had an antiviral effect against HBV infection in PHH. PHHs were infected with AdV encoding N4BP1 to generate N4BP1-overexpressing PHHs 2 days before HBV infection (Fig. 4b), and pgRNA and core-associated HBV DNA levels were decreased in the cells at 14 d.p.i. (Fig. 4c, d).

In RNA sequencing, we set a cutoff value of gene expression at absolute fold change greater than 2 and an adjusted *P*-value less than 0.1 compared with control cells and found 178 genes upregulated in N4BP1-overexpressing vs control PHHs and 230 genes downregulated (Fig.4e f and Table S1). None of these factors were regulatory transcription factors. In addition, pathway enrichment analysis did not show significant coordinated changes in genes associated with translational pathways (Fig. 4g). These findings suggest that N4BP1 overexpression was not associated with overarching changes in the expression of regulatory transcription factors that would also affect HBV RNA production.

### N4BP1 expression is not induced by HBV

**Fig. 5.**
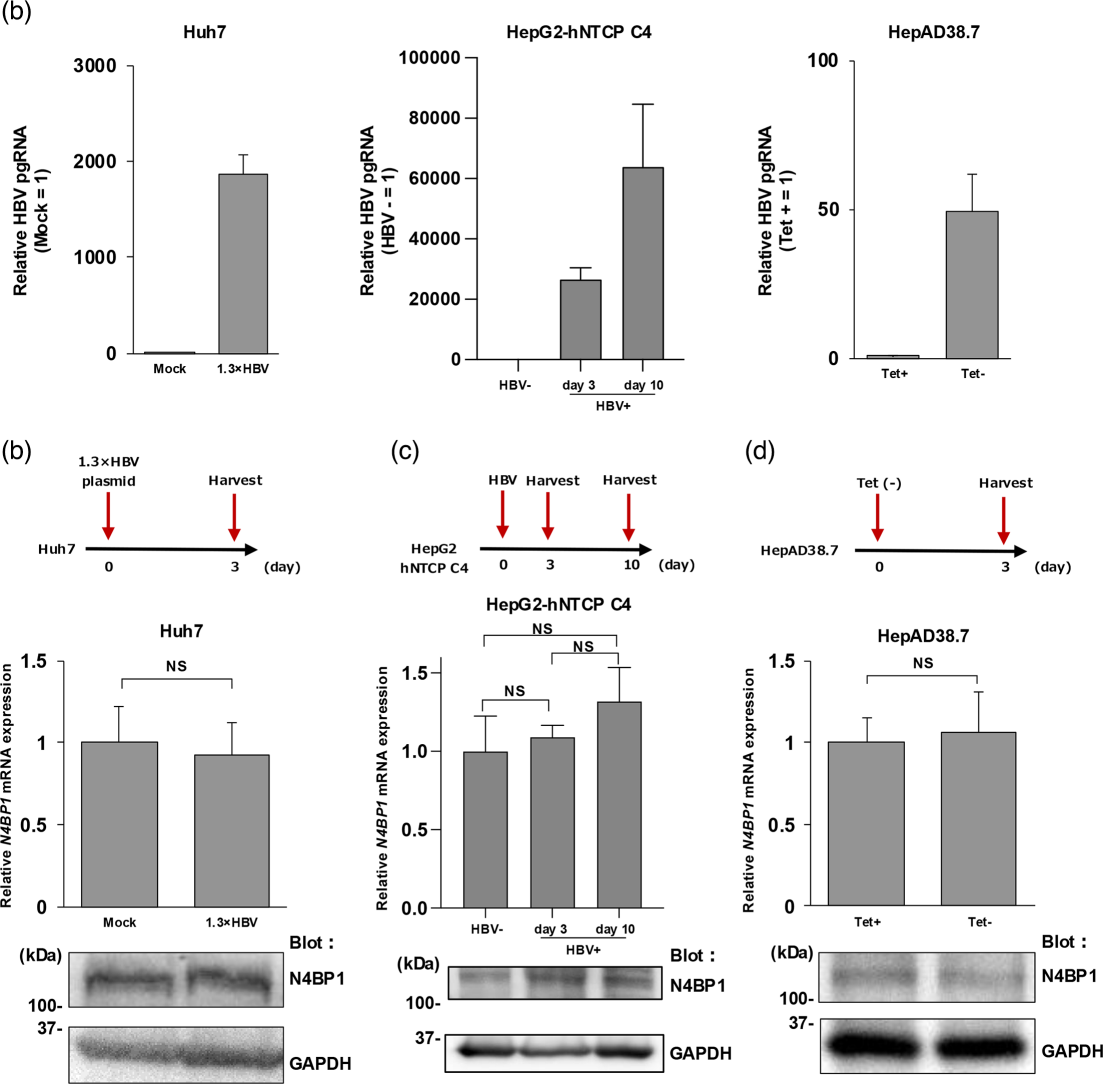
N4BP1 expression is not induced by HBV. (a) We evaluated whether N4BP1 expression is induced by HBV infection or transfection in hepatocyte cell lines. In each system, we confirmed by qPCR whether pgRNA was induced. (b) Huh7 cells were transfected with a plasmid containing the 1.3-fold-overlength genome of HBV genotype C, and cells were collected at 3 d.p.t. (upper). *N4BP1* mRNA expression was assessed by qPCR (middle) and protein expression by WB (lower). (c) HepG2-hNTCP C4 cells were infected with HBV genotype D, and cells were collected at 3 and 10 d.p.i. (upper). N4BP1 expression level by qPCR (middle). N4BP1 expression levels by WB (lower). (d) Tetracycline was removed from the medium of HepAD38.7 cells, and the cells were collected 3 days later (upper). N4BP1 expression levels by qPCR (middle). N4BP1 expression levels by WB (lower). Error bars represent the sd. *P<0.05, **P<0.01, as determined by the Mann–Whitney U test. All qPCR experiments were independently performed at least three times.

We then evaluated whether N4BP1 expression level was increased by HBV infection. A plasmid containing the 1.3-fold-overlength genome of HBV genotype C was transfected into Huh7 cells, and N4BP1 expression was assessed by WB and qPCR at 3 d.p.t. Transfection of the HBV genome did not significantly affect N4BP1 expression at either the transcriptional or protein level (Figs 5a and 4b). N4BP1 expression also did not significantly change in HepG2-hNTCP C4 cells at 3 and 10 d.p.i. (Fig. 5a, c). Finally, we measured N4BP1 expression in HepAD38.7 cells that stably secrete HBV particles unless cultured in the presence of tetracycline. In this system, the removal of tetracycline and the induction of HBV particle production also did not result in any change in N4BP1 expression (Fig. 5a, d). Together, these results indicate that N4BP1 expression is not affected by HBV infection.

### The KH-like and RNase domains of N4BP1 are critical for the anti-HBV effect of N4BP1

**Fig. 6.**
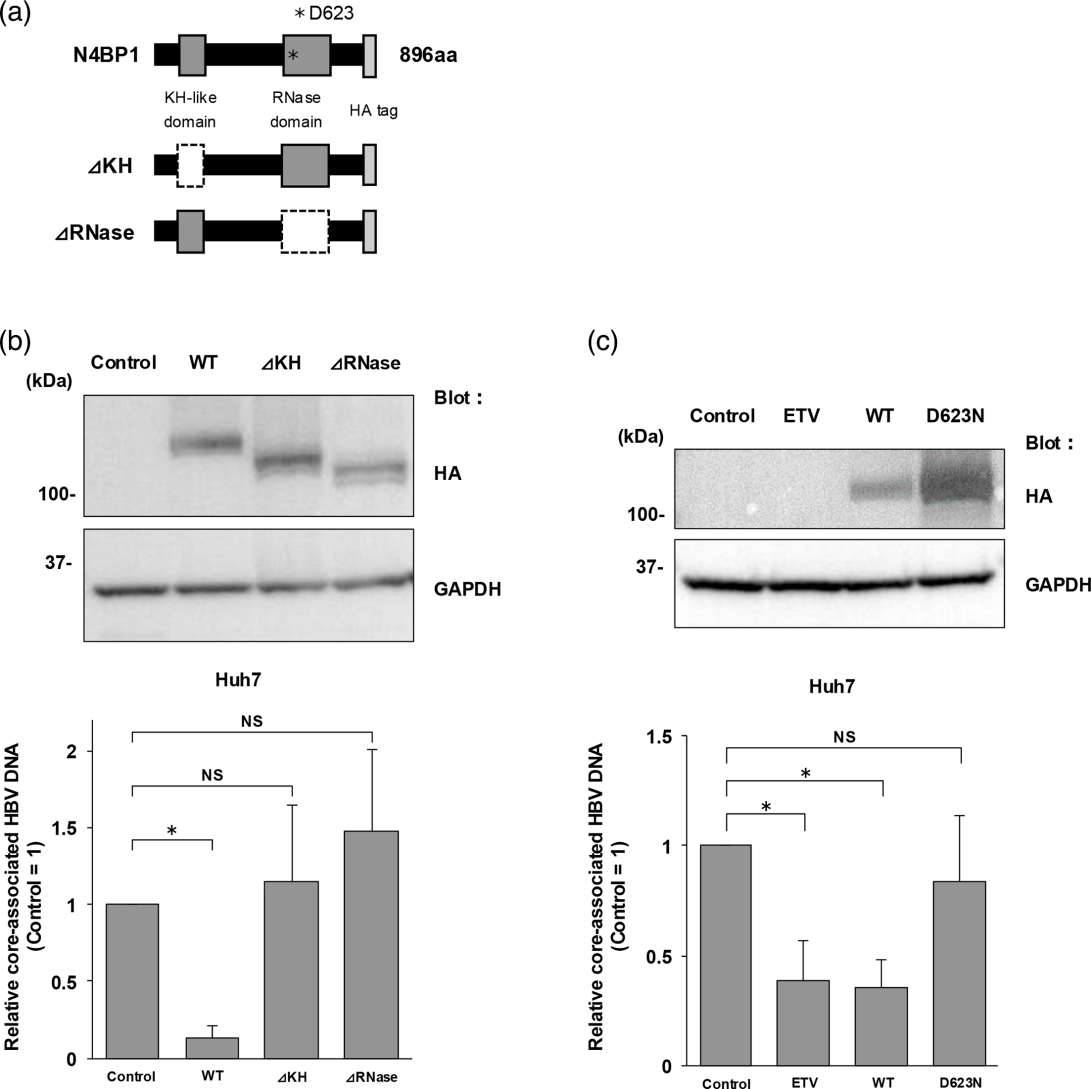
KH-like domain and RNase domain are involved in the anti-HBV effect of N4BP1. (a) Schematic of N4BP1. N4BP1 is composed of 896 aa and has both a KH-like domain and an RNase domain. We constructed a KH-like domain-deficient mutant (deletion of aa 59–143) and an RNase domain-deficient mutant (deletion of aa 617–769). Each plasmid had an HA tag at its C-terminus. (b) Huh7 cells were transfected with the plasmid containing the 1.3-fold-overlength genome of HBV genotype C and each expression plasmid (control vector, N4BP1 WT, ΔKH and ΔRNase), and cells were collected at 3 d.p.t. WB was used to confirm the expression of each N4BP1 mutant (upper, anti-HA tag). GAPDH was used as a loading control (lower, anti-GAPDH). HBV levels were assessed by measuring intracellular core-associated HBV DNA levels at 3 d.p.t. (c) Huh7 cells were transfected with the plasmid containing the 1.3-fold-overlength genome of HBV genotype C and each expression plasmid (control vector, WT and D623N), and cells were collected at 3 d.p.t. WB was used to confirm the expression of each N4BP1 mutant (upper, anti-HA tag). GAPDH was used as a loading control (lower, anti-GAPDH). HBV levels were assessed by measuring intracellular core-associated HBV DNA levels at 3 d.p.t. ETV was used as a positive control. Error bars represent the sd. *P<0.05 as determined by the Mann–Whitney U test. All qPCR experiments were independently performed at least three times.

As shown in Fig. 6(a), N4BP1 is composed of 896 aa and contains a KH-like domain at its N-terminus side and an RNase domain at its C-terminus [34]. To examine which N4BP1 domains are needed to suppress HBV replication, we constructed deletion mutants lacking either the KH-like (ΔKH; deletion of aa59–143) or the RNase domain (ΔRNase; deletion of aa617–769).

The plasmid encoding WT or mutant N4BP1 was respectively co-transfected into naïve Huh7 cells with the plasmid containing the 1.3-fold-overlength genome of HBV genotype C, and the core-associated HBV DNA levels were measured. The expression of the deletion mutants was confirmed by WB using an HA-tagged antibody (Fig. 6b, upper). The core-associated HBV DNA level in N4BP1-overexpressing cells was significantly reduced, while the core-associated HBV DNA levels in the ΔKH- or ΔRNase-expressing cells were comparable to those in the control cells (Fig. 6b, lower).

Residue D623 in the RNase domain of N4BP1 (Fig. 6a) has been shown to be important for the protein’s RNase activity [21]. A D623N substitution causes a loss of RNase activity without changing the structure of N4BP1. Co-transfecting the Huh7 cells with a plasmid expressing this N4BP1 D623 mutant and the HBV overlength genome also did not lead to a decrease in core-associated HBV DNA levels (Fig. 6c). These results suggest that both the KH-like and RNase domains are critical for the anti-HBV effect of N4BP1.

### N4BP1 promotes pgRNA degradation

**Fig. 7.**
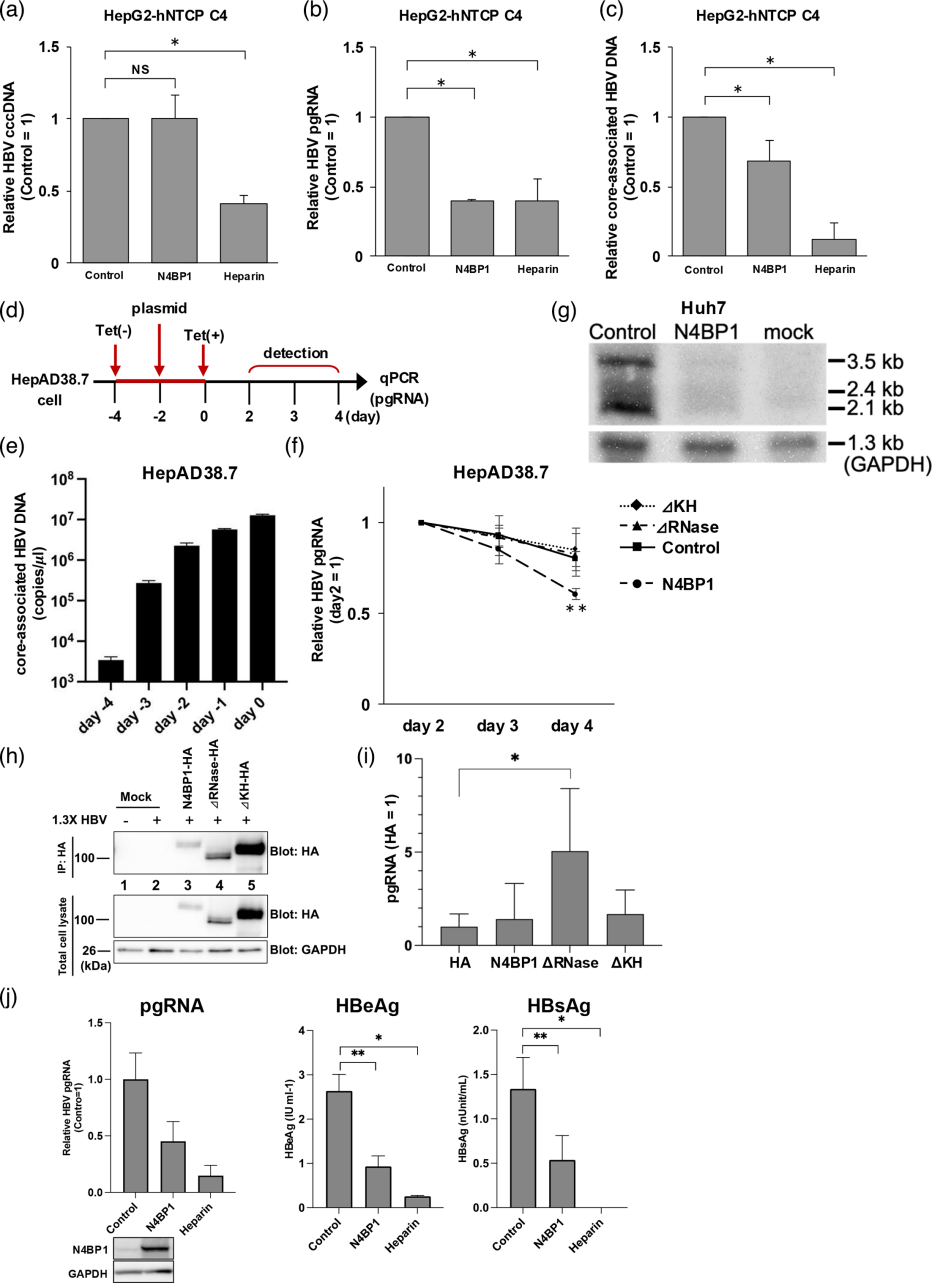
N4BP1 degrades HBV RNAs by binding HBV RNAs. HepG2-hNTCP C4 cells were infected with HBV genotype D, and cells were collected at 14 d.p.i. Comparison of cccDNA (a), pgRNA (b) and core-associated HBV DNA (c) levels in N4BP1-overexpressing HepG2-hNTCP C4 cells and control at 14 d.p.i. Heparin was used as a positive control. (d) Time course of pgRNA degradation assay in HepAD38.7 cells. (e) Core-associated HBV DNA levels in HepAD38.7 cells prior to tetracycline addition. (f) pgRNA levels over time in HepAD38.7 cells overexpressing N4BP1 WT, ΔKH, ΔRNase or control. (g) Northern blotting analyses in control, N4BP1-overexpressing Huh7 cells transfected with the 1.3-fold-overlength genome of HBV and naïve Huh7 cells (mock). (h) Huh7 cells were co-transfected with the 1.3-fold-overlength genome of HBV genotype C with HA control vector, HA-tagged N4BP1 or ΔRNase. Cells were collected at 3 d.p.t. and RNA immunoprecipitation assay was performed. WB was used to confirm the expression of each N4BP1 mutant (blot: HA) and IP was performed. GAPDH was used as a loading control (lower, anti-GAPDH). (i) RNA was extracted from total cell lysate and the immunoprecipitated protein, and then, pgRNA was measured by qPCR. (j) PHHs were infected with HBV, and supernatant was collected at 14 d.p.i. RNA was extracted from the cells and pgRNA was measured, and N4BP1 overexpression was confirmed by WB (left). The viral protein levels in the supernatant were measured by HBeAg CLIA (middle) and HBsAg ELISA (right). Error bars represent the sd. *P<0.05, **P<0.01, as determined by the Mann–Whitney U test. All experiments were independently performed at least three times.

Having shown the effect of N4BP1 on core-associated HBV DNA, we then sought to determine more specifically which step of HBV replication N4BP1 suppresses. To that end, we assessed the impact of N4BP1 overexpression on cccDNA and pgRNA levels. HepG2-hNTCP C4 cells were infected with HBV, and cells were harvested at 14 d.p.i. While cccDNA levels were not affected by the overexpression of N4BP1, pgRNA and core-associated HBV DNA levels were both significantly reduced (Fig. 7a–c). The HBV progeny secreted from infected HepG2-hNTCP C4 cells is not infectious [35,36], and thus, only single-round infection is observed in this system. Thus, these results and the data of the promoter assay (Fig. 4a) indicate that N4BP1 affects the steps of HBV replication after cccDNA was formed and decreases pgRNA.

To elucidate the mechanism of pgRNA suppression by N4BP1, we turned to HepAD38.7 cells. First, the cells were cultured for 2 days in media without tetracycline to induce HBV replication. Then, N4BP1 or the domain deletion mutant plasmids were transfected, and tetracycline was added at 2 d.p.t. to stop viral replication (Fig. 7d). Until tetracycline addition, core-associated HBV DNA was measured to confirm HBV replicated sufficiently (Fig. 7e). Then, cells were collected starting 2 days after adding tetracycline, to measure the pgRNA levels (Fig. 7f). As shown in Fig. 7(f), the expression of WT N4BP1 augmented the rate of pgRNA degradation. In contrast, both domain-deficient mutants resulted in pgRNA degradation at a rate similar to the control, suggesting that N4BP1 affects the degradation rate of pgRNA. Next, we examined whether N4BP1 expression also suppresses HBV RNAs other than pgRNA. We co-transfected Huh7 cells with a plasmid expressing N4BP1 and a plasmid containing 1.3-fold-overlength genomes of HBV genotype C. At 3 d.p.t., cells were collected and HBV RNA expression was measured by Northern blotting. Results showed that control Huh7 cells showed intense expression of 3.5 and 2.4/2.1 kb HBV RNA, whereas N4BP1 overexpression Huh7 cells showed weak expression of all these RNAs (Fig. 7g).

Next, to test that N4BP1 binds HBV RNA directly, we performed an RNA immunoprecipitation (RIP) assay. Huh7 cells were co-transfected with a plasmid containing the 1.3-fold-overlength genome of HBV genotype C and HA-tagged N4BP1 WT or domain-deficient mutants or HA control vector. Cells were collected at 3 d.p.t. and immunoprecipitated with anti-HA-tag monoclonal antibody magnetic beads. WT and N4BP1 domain-deficient mutants were confirmed to be precipitated by an anti-HA-tag monoclonal antibody by WB (Fig. 7h). The RNA–protein interaction was expected to be most stable in the ΔRNase domain mutant, and our RIP-qPCR assay confirmed this (Fig. 7i). In contrast, the amount of pgRNA recovered with WT N4BP1 and ΔKH-like domain mutants was far less, presumably due to the intact RNase activity for WT N4BP1 and reduction of binding affinity for ΔKH-like domain mutants.

Furthermore, to investigate whether N4BP1 also led to reduced expression of viral proteins, we assessed levels of HBeAg and HBsAg in the supernatant of HBV-infected PHHs at 14 d.p.i., the timepoint when we had observed decreased pgRNA (Fig. 7j). These HBV proteins we evaluated were decreased in the N4BP1-overexpressing PHHs compared with control, indicating that N4BP1 inhibits PreS2/S RNA and pgRNA, consistent with our northern blot data.

### N4BP1 expression is induced by IFN-*α*/*λ* in PHHs

**Fig. 8.**
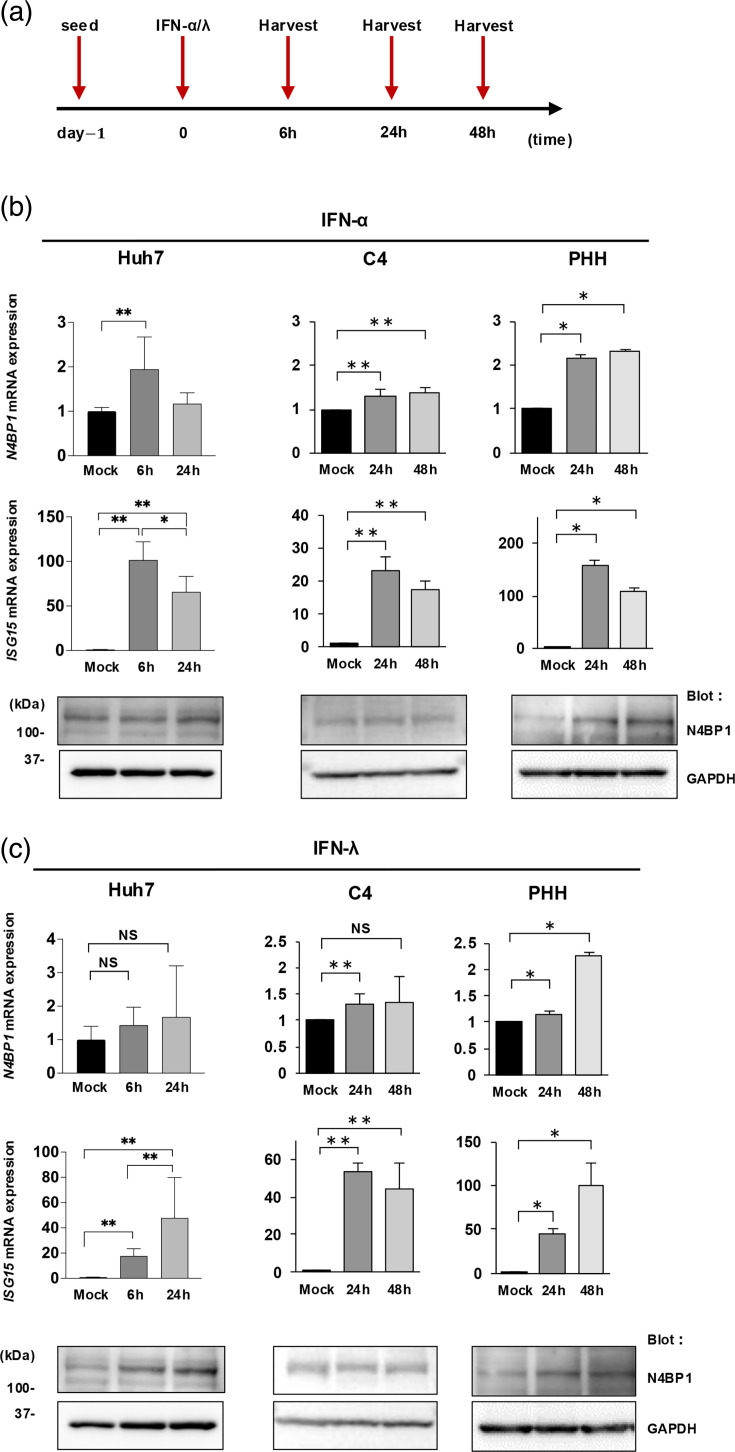
N4BP1 expression is induced by IFN-α and IFN-λ in PHHs. (a) Time course of the drug treatment assay. Cells were harvested at 6, 24 or 48 h post-IFN-*α*/*λ* treatment. (b) N4BP1 expression levels by qPCR in Huh7 cells, HepG2-hNTCP C4 cells and PHHs treated with IFN-*α* (upper). ISG15 expression levels by qPCR (middle). N4BP1 protein expression levels by WB. GAPDH was used as a loading control (lower). (c) N4BP1 expression levels by qPCR in Huh7 cells, HepG2-hNTCP C4 cells and PHHs treated with IFN-*λ* (upper). ISG15 expression levels by qPCR (middle). N4BP1 protein expression levels by WB. GAPDH was measured as a loading control (lower).

It has been reported that N4BP1 expression is increased three- to fivefold with IFN-*α* treatment in Jurkat cells and THP-1 cells [21]. To see if this was the case in PHHs and hepatoma cell lines, we treated Huh7, HepG2-hNTCP C4 and PHHs with 1,000 U ml^−1^ of IFN-*α* and 100 ng ml^−1^ of IFN-*λ*, which are known to also inhibit HBV replication. Cells were collected at 6, 24 and 48 h post-drug treatment without HBV infection, and the expression of *N4BP1* mRNA and protein was examined by qPCR and WB, respectively (Fig. 8a). *ISG15* mRNA was included as a positive control. As shown in Fig. 8(b), levels of *N4BP1* mRNA were increased twofold in both Huh7 and HepG2-hNTCP C4 cells by IFN-*α* at 6 and 24 h, respectively, which is small, albeit significant in expression. IFN-*λ* stimulation increased levels of *N4BP1* mRNA in PHHs at 24 and 48 h significantly, and N4BP1 protein levels were induced in Huh7 cells and PHHs (Fig. 8c).

**Fig. 9.**
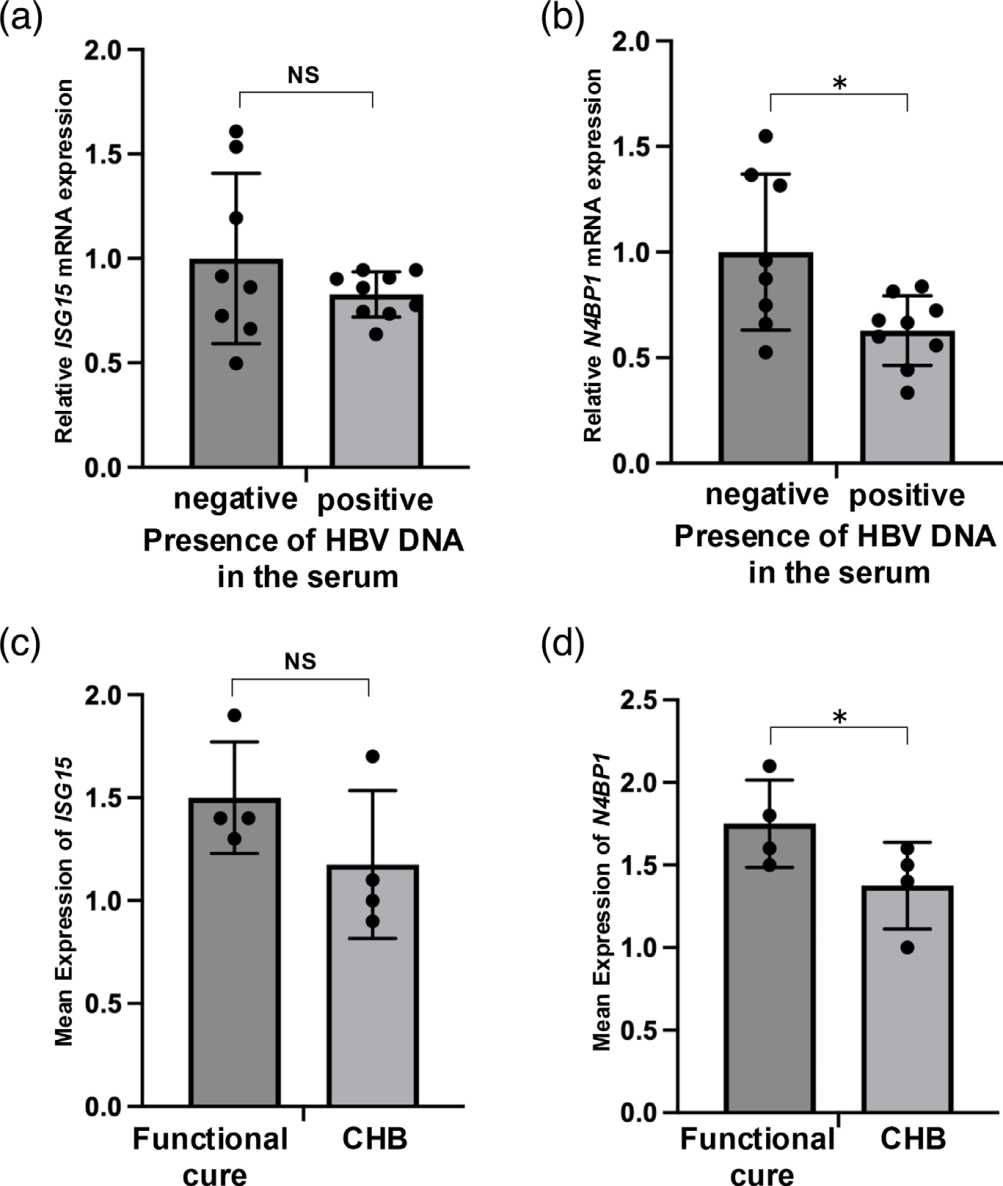
ISG15 and N4BP1 expression levels in HBV-infected human liver tissues and in scRNA-seq database of patients with CHB. The mRNA levels of *ISG15* (a) and *N4BP1* (b) from non-cancerous tissues of HBV-HCC patients with HBs-Ag (+), HBe-Ag (−) and HBe-Ab (+) were examined by qPCR. The samples were divided into HBV DNA-positive group (*n*=9) and HBV DNA-negative group (*n*=8). The average mRNA levels of *ISG15* (c) and *N4BP1* (d) in their respective positive hepatocytes from functional cure patients (*n*=4) or patients with CHB (*n*=4) in scRNA-seq database [27]. Error bars represent the sd. *P<0.05, as determined by the Mann–Whitney U test.

IFN-*α* and IFN-*λ* treatment in PHHs resulted in an approximately twofold increase in *N4BP1* mRNA expression, and protein level was also increased. Collectively, these results indicate that IFN-*α* treatment induces N4BP1 expression in Huh7 and HepG2-hNTCP C4 cells, while both IFN-*α* and IFN-*λ* induce N4BP1 expression in PHHs. As primary cells, PHHs are more physiologically relevant than hepatoma lines such as Huh7 and HepG2-hNTCP C4 cells in which the response to IFN is disrupted. To build upon our *in vitro* findings, we then examined samples from patients with HBV-HCC and publicly available scRNA-seq data obtained from liver biopsies of patients with CHB and functional cure to assess whether there was an association between *N4BP1* mRNA expression in the liver and HBV reactivation status. For HCC resection samples, all the patients showed HBs-Ag (+), HBe-Ag (−) and HBe-Ab (+) and were categorized as either HBV DNA positive or negative per HBV DNA levels in the serum, and *ISG15* and *N4BP1* mRNA expressions were assayed in non-cancerous liver tissue from these patients (Fig. 9a, b). *ISG15* mRNA did not significantly differ between the patient groups, but *N4BP1* mRNA was significantly higher in patients who were HBV DNA negative vs positive.

Consistent with these data, scRNA-seq data also indicated that the average *N4BP1* mRNA expression in their positive cells was significantly higher in patients with functional cure compared with CHB (Fig. 9c, d).

## Discussion

NAs are generally well tolerated and very effective in inhibiting HBV replication and are currently the most commonly used treatment for chronic hepatitis B. However, since NAs inhibit reverse transcription, HBV cccDNA remains in the nucleus of hepatocytes, so the patient is never completely cured. Indeed, patients who discontinue NA treatment can experience virologic relapse and thus must continue NA therapy for extended periods. Also, long-term treatment with NA is associated with the development of antiviral drug resistance [37,39] although current common NAs such as ETV and tenofovir showed low frequency of drug resistance. Therefore, it is required to develop new HBV treatments that cure hepatitis B to prevent the need for long-term treatment with NA. Thus, we sought to identify host factors involved in HBV proliferation that would be distinct from the viral sites targeted by NAs. In a library of 132 RBPs with known RNA-binding domains such as RNA recognition motif domains or zinc finger domains, we found that N4BP1 inhibited HBV replication, was negatively correlated with HBV core-associated HBV DNA levels in hepatoma cell lines and suppressed HBV replication.

We found that both the KH-like domain and the RNase domain of N4BP1 were essential for the protein’s anti-HBV effect (Fig. 6b). It has been reported that the KH-like domain binds to RNA or ssDNA and is involved in splicing, transcriptional regulation and translational regulation [34,40, 41]. On the other hand, the RNase domain of N4BP1 is also involved in regulating genome stability and repressing the expression of target genes through mRNA strand breaks [42]. Based on our RIP assay data, it is possible that N4BP1 binds HBV RNA via its KH-like domain and degrades HBV RNA by the RNase activity of its RNase domain. However, precise mechanisms of how N4BP1 recognizes and binds to HBV RNAs remain to be clarified. This would need to be confirmed with an RIP assay using a doubly deficient RNase and KH-like domain mutant. In fact, in this study, N4BP1 suppressed HBV replication by interfering with pgRNA in the HBV life cycle (Fig. 7). We also found that N4BP1 suppresses not only the 3.5 kb RNA of HBV but also the 2.4/2.1 kb RNA by northern blot, which suggests that N4BP1 promotes the degradation of 3.5 and 2.4/2.1 kb HBV RNAs or suppresses HBV RNA production. However, it is still unclear and remains to be assessed whether N4BP1 promotes the degradation of HBV RNAs and/or suppresses RNA production and whether HBx is also affected by N4BP1.

N4BP1 is known to be an inhibitor of innate immunity and inflammation-mediated cytokine production. NF*κ*B is an important transcription factor for the elicitation of cytokine responses, and its activation is necessary for many immune responses. N4BP1 interacts with the NF*κ*B signal essential modulator (NEMO, also known as I*κ*B kinase *γ*) to suppress Toll-like receptor (TLR)-dependent NFκB activation [43]. Furthermore, even *in vivo*, it has been reported that N4BP1-/- mice develop mild inflammation and have exacerbated TLR-dependent inflammatory responses [44]. Recently, N4BP1 was found as a new hotspot gene for HBV integration [45]. Although the significance of HBV integration to N4BP1 remains unknown, it is interesting that the virus integration occurred on the N4BP1 gene locus, which has anti-HBV activity. Regarding virus replication, N4BP1 is known to directly recognize and degrade retroviruses such as HIV-1 [21] and to be involved in the replication of the porcine reproductive and respiratory syndrome virus [46]. In both cases, the RNase activity of N4BP1 was found to inhibit viral replication by directly degrading viral RNA, consistent with our data. In addition, the RNase domain of N4BP1 has a similar structure to that of MCPIP1 [47], which has been reported to recognize and degrade HBV pgRNA directly [15]. However, MCPIP1 is a cytoplasmic RNase, whereas N4BP1 is mainly localized in the nucleus. Furthermore, MCPIP1 has not been reported to suppress 2.4 and 2.1 kb HBV RNAs, whereas we have demonstrated that N4BP1 suppresses these RNAs. These results suggest that MCPIP1 and N4BP1 may regulate HBV-degrading mRNAs through different processes. The mechanism behind our finding that the KH-like domain of N4BP1 was also important for the anti-HBV effect of this protein is presumably due to its binding to HBV RNA, but further work is needed to elucidate the mechanism more precisely.

Although N4BP1 expression has been reported to be induced by type I IFN [21], the stimulation of N4BP1 expression by IFN-*α* (type I IFN) and IFN-*λ* (type III IFN) treatment varied between the PHHs and hepatoma cell lines we tested. This could be due to the disrupted IFN signalling reported in the cell lines Huh7 and HepG2 that result in them being less sensitive to IFN [48,49]. Also, ISG15 is an inducible factor by IFN-*α* and IFN-*λ* [50,51]. In characterizing N4BP1 expression, we found that N4BP1 expression was not induced by HBV infection or transfection in hepatoma cell lines (Fig. 5). Furthermore, in clinical liver tissue samples, N4BP1 expression was significantly lower in HBV-DNA-positive vs HBV-DNA-negative samples (Fig. 9b). In addition, scRNA-seq data indicated that N4BP level in functional cure patients was higher than that in patients with CHB (Fig. 9d). These results suggest that N4BP1 expression may differ according to each individual and that the expression of N4BP1 in the liver may impact HBV reactivation. Otherwise, it is thought that factors upstream of N4BP1 may cause differences in N4BP1 expression and affect HBV reactivation. In addition, research has shown that N4BP1 can be inactivated by caspase-8 [44] or mucosa-associated lymphoid tissue lymphoma translocation 1 (MALT1) [21] in immune cells. In the latter instance, N4BP1 inactivation by MALT1 induced HIV-1 reactivation. This does not appear to be the case in our study, as our RNA-seq data did not indicate MALT1 induction by N4BP1 overexpression in PHH, but other signalling proteins may have a similar effect, resulting in greater HBV replication in hepatocytes. This will need to be studied further.

A limitation of our current study is the small number of patient samples used to evaluate the relationship between N4BP1 and HBV reactivation. Studies with a greater number of samples are needed to further characterize this possible relationship. In addition, although the HBV promoter assay suggested that N4BP1 did not inhibit all HBV promoters and transcriptomic analysis implies that N4BP1 was not associated with transcriptional regulation, it is required to strengthen these results by other methods such as ActD chase assay. Also, the identification of the element(s) of HBV RNA that bind N4BP1 remains to be determined. As HBV RNAs overlap each other, explicit identification of the binding region(s) was difficult. A previous report suggested that sequence motif(s) other than GC dinucleotides may be recognized by N4BP1 as N4BP1 can degrade HIV-1 transcripts, which have a markedly reduced frequency of GC dinucleotides [21]. However, based on our data, it is conceivable that an overlapping area or areas of the 3.5 and 2.4 kb/2.1 kb HBV RNAs may bind to N4BP1.

In conclusion, to investigate different treatments against HBV infection from currently approved HBV therapeutics, we focused on RBPs. We found that N4BP1 is an RBP with novel anti-HBV activity, inhibiting HBV replication via degradation of HBV RNAs. Although the detailed molecular mechanism behind the anti-HBV effect of N4BP1 remains to be uncovered, understanding the association between N4BP1 and HBV could contribute to the development of new HBV treatments.

## Supplementary material

10.1099/jgv.0.002082 Uncited Supplementary Material 1.

## Abbreviations

AdV: adenovirus vector
cccDNA: covalently closed circular DNA
CHB: chronic HBV infection
CLIA: Chemiluminescence Immunoassay
DMEM: Dulbecco's Modified Eagle Medium
d.p.i.: days post-infection
d.p.t.: days post-transfection
ETV: entecavir
GEq: genome equivalents
HBV: hepatitis B virus
HCC: hepatocellular carcinoma
ISG: IFN-stimulated gene
KO: knockout
MALT1: mucosa-associated lymphoid tissue lymphoma translocation 1
NA: nucleos(t)ide analogue
N4BP1: NEDD4-binding protein 1
N4BP1 AdV: N4BP1-expressing AdV
NTCP: sodium-taurocholate cotransporting polypeptide
PEG: polyethylene glycol
pgRNA: pregenomic RNA
PHH: primary human hepatocyte
qPCR: quantitative PCR
RBP: RNA-binding protein
rcDNA: relaxed circular DNA
RIP assay: RNA immunoprecipitation assay
RT-qPCR: reverse transcription qPCR
TLR: Toll-like receptor
WB: western blotting
