# Supplementary material for: NEDD4-binding protein 1 suppresses hepatitis B virus replication by regulating viral RNAs
Source: J Gen Virol. 2025 Mar 20;106(3):002082. doi: 10.1099/jgv.0.002082 (PMC12282332; doi:10.1099/jgv.0.002082)
Supplement: Uncited Supplementary Material 1. [file jgv-106-02082-s001.pdf]

S1 Table Differential gene expression profile by RNA-seq of HBV-infected PHHs overexpressing N4BP1 compared to HBV-infected control PHHs.

Factor name	baseMean	log2FoldChange	lfcSE	stat	pvalue	padj	distance	significance
N4BP1	11471.14	4.223	0.081	51.88	0.00E+00	0.00E+00	Inf	Upregulated
NNMT	1189.55	3.009	0.137	21.94	1.08E-106	6.59E-103	102.226	Upregulated
FGB	70363.60	1.589	0.073	21.79	3.14E-105	1.28E-101	100.905	Upregulated
KRT8	27810.42	1.444	0.071	20.27	2.14E-91	6.55E-88	87.196	Upregulated
FSTL1	2342.94	1.859	0.097	19.13	1.34E-81	3.27E-78	77.507	Upregulated
KRT18	11689.26	1.372	0.078	17.48	2.03E-68	4.14E-65	64.398	Upregulated
FGG	74088.49	1.262	0.075	16.84	1.15E-63	2.01E-60	59.710	Upregulated
SAA2	811.77	2.184	0.132	16.55	1.49E-61	2.02E-58	57.736	Upregulated
SAA1	4684.75	1.545	0.100	15.53	2.19E-54	2.43E-51	50.638	Upregulated
ANGPTL4	6260.94	1.639	0.106	15.48	4.95E-54	5.04E-51	50.324	Upregulated
MT1E	441.04	2.482	0.163	15.27	1.22E-52	1.15E-49	49.002	Upregulated
CCND1	1089.74	1.818	0.123	14.76	2.57E-49	2.24E-46	45.686	Upregulated
FGA	120757.14	1.129	0.079	14.34	1.27E-46	9.15E-44	43.053	Upregulated
BCL2L1	2946.48	1.145	0.085	13.49	1.70E-41	1.16E-38	37.954	Upregulated
KRT7	871.67	3.792	0.286	13.25	4.38E-40	2.81E-37	36.747	Upregulated
ID1	2106.95	1.333	0.101	13.21	7.19E-40	4.39E-37	36.382	Upregulated
EPAS1	3080.23	1.151	0.088	13.10	3.22E-39	1.87E-36	35.746	Upregulated
CCND2	520.95	1.809	0.145	12.48	9.81E-36	5.00E-33	32.352	Upregulated
TRNP1	1915.79	1.271	0.103	12.36	4.06E-35	1.98E-32	31.728	Upregulated
C10orf54	611.04	1.596	0.133	12.02	2.74E-33	1.29E-30	29.933	Upregulated
SYT12	324.54	2.271	0.190	11.92	9.36E-33	4.09E-30	29.476	Upregulated
AHNAK2	226.50	2.465	0.215	11.49	1.43E-30	5.15E-28	27.399	Upregulated
SLC2A2	2432.96	1.233	0.108	11.45	2.45E-30	8.56E-28	27.095	Upregulated
SLC35C1	2293.29	1.037	0.093	11.18	5.01E-29	1.70E-26	25.790	Upregulated
ANXA3	372.44	2.157	0.194	11.12	9.99E-29	3.21E-26	25.584	Upregulated
NBEAL2	597.84	1.550	0.140	11.09	1.35E-28	4.23E-26	25.421	Upregulated
S100A11	1740.92	1.150	0.108	10.69	1.08E-26	2.88E-24	23.569	Upregulated
MT2A	1497.14	2.668	0.250	10.66	1.60E-26	4.17E-24	23.532	Upregulated
NR0B2	164.15	2.731	0.258	10.58	3.70E-26	9.18E-24	23.199	Upregulated
SLC7A7	413.42	1.635	0.155	10.58	3.75E-26	9.18E-24	23.095	Upregulated
CPA4	137.93	2.983	0.290	10.27	9.64E-25	1.90E-22	21.925	Upregulated
GALK1	964.70	1.216	0.118	10.29	8.10E-25	1.65E-22	21.816	Upregulated
MYL9	303.84	2.131	0.208	10.24	1.33E-24	2.49E-22	21.708	Upregulated
DUSP6	275.19	1.954	0.193	10.11	4.84E-24	8.84E-22	21.144	Upregulated

MCAM	494.51	1.515	0.154	9.83	8.21E-23	1.30E-20	19.942	Upregulated
TAT	3852.07	1.038	0.106	9.81	1.00E-22	1.57E-20	19.832	Upregulated
ABALON	1090.24	1.040	0.107	9.68	3.58E-22	5.40E-20	19.295	Upregulated
CUX2	559.43	1.362	0.142	9.62	6.81E-22	9.79E-20	19.058	Upregulated
EPHA2	922.79	1.075	0.113	9.51	1.89E-21	2.65E-19	18.608	Upregulated
MFSD2A	185.09	2.400	0.254	9.44	3.57E-21	4.96E-19	18.462	Upregulated
MT1X	162.38	2.511	0.269	9.33	1.06E-20	1.43E-18	18.022	Upregulated
LOXL4	660.17	1.445	0.155	9.32	1.19E-20	1.58E-18	17.861	Upregulated
TAGLN	208.81	2.002	0.221	9.06	1.30E-19	1.63E-17	16.906	Upregulated
TGM2	3127.81	2.409	0.268	8.99	2.37E-19	2.92E-17	16.709	Upregulated
SDC4	2311.64	1.028	0.115	8.95	3.54E-19	4.28E-17	16.401	Upregulated
NAT8	402.48	1.372	0.155	8.85	8.88E-19	1.05E-16	16.036	Upregulated
SLC25A25	396.24	1.399	0.158	8.84	9.94E-19	1.17E-16	15.994	Upregulated
UNC5CL	429.19	1.305	0.155	8.44	3.29E-17	3.47E-15	14.519	Upregulated
ETV1	291.18	1.503	0.178	8.42	3.79E-17	3.93E-15	14.484	Upregulated
CNN2	908.97	1.197	0.144	8.29	1.09E-16	1.08E-14	14.018	Upregulated
IL18	136.71	2.384	0.289	8.24	1.68E-16	1.61E-14	13.997	Upregulated
ETV5	105.02	2.531	0.314	8.05	8.01E-16	7.14E-14	13.388	Upregulated
TGFB2	165.15	2.003	0.255	7.85	4.30E-15	3.58E-13	12.606	Upregulated
MT1G	134.20	2.275	0.300	7.59	3.28E-14	2.54E-12	11.817	Upregulated
MYOF	456.37	1.170	0.155	7.56	3.88E-14	2.93E-12	11.593	Upregulated
SDS	47.14	4.159	0.581	7.16	7.86E-13	4.98E-11	11.111	Upregulated
SPRY4	75.58	2.673	0.367	7.29	3.09E-13	2.07E-11	11.014	Upregulated
FBLIM1	283.70	1.294	0.179	7.21	5.52E-13	3.57E-11	10.527	Upregulated
SLC16A12	214.82	1.540	0.221	6.98	2.95E-12	1.68E-10	9.895	Upregulated
CRP	80.82	2.486	0.363	6.85	7.28E-12	3.94E-10	9.728	Upregulated
FLNC	174.91	1.541	0.223	6.92	4.47E-12	2.49E-10	9.726	Upregulated
RBMS2	347.10	1.124	0.163	6.91	4.89E-12	2.69E-10	9.636	Upregulated
DEFB1	431.44	1.021	0.152	6.71	1.98E-11	1.01E-09	9.055	Upregulated
THBS1	348.31	1.283	0.193	6.65	3.01E-11	1.46E-09	8.930	Upregulated
ATP2B4	550.46	1.016	0.155	6.54	6.00E-11	2.74E-09	8.623	Upregulated
SH3YL1	385.54	1.027	0.158	6.49	8.72E-11	3.87E-09	8.474	Upregulated
RNF157	97.55	1.911	0.298	6.40	1.51E-10	6.43E-09	8.412	Upregulated
CCAT1	62.61	2.672	0.427	6.26	3.77E-10	1.53E-08	8.259	Upregulated
PRICKLE2	216.34	1.221	0.200	6.10	1.08E-09	4.13E-08	7.484	Upregulated
KRT80	29.18	4.207	0.753	5.59	2.31E-08	6.78E-07	7.467	Upregulated
LBH	123.98	1.881	0.315	5.97	2.32E-09	8.28E-08	7.328	Upregulated
ATOH8	256.52	1.110	0.185	6.02	1.77E-09	6.45E-08	7.276	Upregulated

FAM105A	94.06	1.734	0.301	5.77	8.05E-09	2.60E-07	6.809	Upregulated
C9	102.29	1.668	0.291	5.73	1.03E-08	3.25E-07	6.699	Upregulated
SVEP1	67.11	2.093	0.370	5.65	1.58E-08	4.80E-07	6.656	Upregulated
PMEPA1	92.30	1.973	0.353	5.60	2.20E-08	6.48E-07	6.496	Upregulated
PDE11A	122.01	1.478	0.265	5.57	2.51E-08	7.33E-07	6.311	Upregulated
LINC01488	91.81	1.755	0.320	5.48	4.21E-08	1.18E-06	6.183	Upregulated
UBASH3B	64.86	2.178	0.404	5.39	7.02E-08	1.87E-06	6.129	Upregulated
BTBD6	273.76	1.031	0.189	5.46	4.74E-08	1.31E-06	5.974	Upregulated
KNDC1	27.44	3.319	0.660	5.03	4.97E-07	1.10E-05	5.969	Upregulated
BCO2	186.65	1.142	0.212	5.38	7.35E-08	1.94E-06	5.824	Upregulated
TMEM147	217.10	1.065	0.198	5.38	7.27E-08	1.93E-06	5.814	Upregulated
MMD	154.96	1.245	0.234	5.33	1.00E-07	2.55E-06	5.731	Upregulated
EFHD1	171.44	1.161	0.229	5.08	3.77E-07	8.61E-06	5.197	Upregulated
MMP24	107.54	1.405	0.282	4.99	6.14E-07	1.32E-05	5.077	Upregulated
HAMP	3098.72	1.062	0.213	4.99	5.95E-07	1.29E-05	5.004	Upregulated
MT1L	68.11	1.734	0.361	4.81	1.55E-06	3.01E-05	4.843	Upregulated
COTL1	89.32	1.466	0.302	4.85	1.23E-06	2.45E-05	4.838	Upregulated
BTBD11	205.61	1.066	0.217	4.91	9.34E-07	1.94E-05	4.832	Upregulated
UBALD2	139.49	1.241	0.254	4.88	1.06E-06	2.16E-05	4.828	Upregulated
SLC25A18	97.07	1.433	0.297	4.83	1.38E-06	2.72E-05	4.786	Upregulated
IP6K3	102.80	1.332	0.286	4.66	3.23E-06	5.80E-05	4.441	Upregulated
ITGB6	117.77	1.287	0.279	4.62	3.90E-06	6.78E-05	4.363	Upregulated
LINC00152	96.06	1.393	0.307	4.54	5.68E-06	9.58E-05	4.253	Upregulated
TSPAN15	113.04	1.218	0.269	4.52	6.05E-06	1.02E-04	4.175	Upregulated
FGFR1	163.44	1.081	0.238	4.54	5.51E-06	9.37E-05	4.171	Upregulated
KDELR3	47.65	1.869	0.428	4.37	1.25E-05	1.96E-04	4.152	Upregulated
MT1F	27.19	2.434	0.622	3.92	9.02E-05	1.08E-03	3.836	Upregulated
UNC5B	91.33	1.369	0.321	4.27	1.99E-05	2.93E-04	3.790	Upregulated
VCAN	78.84	1.463	0.348	4.20	2.67E-05	3.78E-04	3.722	Upregulated
ICOSLG	118.78	1.117	0.264	4.24	2.25E-05	3.24E-04	3.664	Upregulated
FNDC5	63.38	1.537	0.375	4.10	4.20E-05	5.62E-04	3.596	Upregulated
DNAH5	79.09	1.400	0.340	4.12	3.86E-05	5.20E-04	3.570	Upregulated
PLSCR3	82.33	1.480	0.362	4.09	4.30E-05	5.74E-04	3.563	Upregulated
NEXN	103.30	1.190	0.287	4.15	3.38E-05	4.64E-04	3.539	Upregulated
AKAP12	1086.42	1.076	0.261	4.12	3.73E-05	5.06E-04	3.467	Upregulated
TSPAN18	50.89	1.620	0.407	3.98	6.95E-05	8.66E-04	3.465	Upregulated
CMTM3	94.32	1.173	0.291	4.03	5.54E-05	7.09E-04	3.361	Upregulated
ACTA1	31.44	2.012	0.539	3.73	1.91E-04	2.08E-03	3.353	Upregulated

ABLIM2	33.93	1.881	0.500	3.76	1.70E-04	1.88E-03	3.311	Upregulated
MYADM	132.00	1.026	0.255	4.03	5.68E-05	7.26E-04	3.303	Upregulated
TPM4	2732.49	1.054	0.264	4.00	6.41E-05	8.10E-04	3.266	Upregulated
S100A9	75.85	1.293	0.331	3.90	9.45E-05	1.13E-03	3.218	Upregulated
C2orf88	114.79	1.045	0.264	3.96	7.50E-05	9.24E-04	3.209	Upregulated
TEAD4	63.63	1.371	0.356	3.85	1.16E-04	1.35E-03	3.180	Upregulated
TMEM54	107.55	1.053	0.273	3.86	1.15E-04	1.34E-03	3.059	Upregulated
FSCN1	98.81	1.148	0.303	3.79	1.52E-04	1.71E-03	2.997	Upregulated
CCDC113	32.43	1.798	0.518	3.47	5.15E-04	4.81E-03	2.933	Upregulated
CDR2L	66.87	1.308	0.355	3.68	2.33E-04	2.49E-03	2.914	Upregulated
MAGI2-AS3	71.61	1.222	0.333	3.67	2.39E-04	2.54E-03	2.868	Upregulated
SOBP	97.56	1.087	0.294	3.70	2.16E-04	2.32E-03	2.850	Upregulated
TMOD2	79.35	1.186	0.326	3.63	2.81E-04	2.90E-03	2.801	Upregulated
GRAMD1B	71.87	1.205	0.334	3.61	3.11E-04	3.15E-03	2.777	Upregulated
SDC3	100.81	1.041	0.286	3.63	2.81E-04	2.90E-03	2.742	Upregulated
TESC	62.88	1.263	0.364	3.48	5.10E-04	4.77E-03	2.643	Upregulated
TMEM154	49.41	1.376	0.403	3.41	6.46E-04	5.78E-03	2.627	Upregulated
WSCD1	75.10	1.159	0.335	3.46	5.49E-04	5.07E-03	2.571	Upregulated
GDPD5	36.42	1.556	0.488	3.19	1.44E-03	1.11E-02	2.497	Upregulated
PDGFB	29.69	1.631	0.525	3.11	1.88E-03	1.38E-02	2.474	Upregulated
TBX15	66.37	1.189	0.356	3.34	8.42E-04	7.16E-03	2.453	Upregulated
TMEM231	27.19	1.672	0.549	3.05	2.32E-03	1.64E-02	2.447	Upregulated
GMPR	88.08	1.028	0.303	3.39	7.00E-04	6.17E-03	2.437	Upregulated
ASPG	28.44	1.616	0.531	3.04	2.33E-03	1.64E-02	2.409	Upregulated
SYTL2	37.93	1.434	0.458	3.13	1.75E-03	1.30E-02	2.368	Upregulated
HID1	77.61	1.053	0.318	3.31	9.40E-04	7.83E-03	2.355	Upregulated
NT5DC2	40.17	1.408	0.454	3.10	1.93E-03	1.41E-02	2.325	Upregulated
NPM3	59.14	1.194	0.371	3.22	1.30E-03	1.02E-02	2.320	Upregulated
SFN	27.94	1.582	0.538	2.94	3.26E-03	2.14E-02	2.300	Upregulated
GLIPR2	52.65	1.229	0.395	3.12	1.83E-03	1.35E-02	2.237	Upregulated
CHST3	53.65	1.204	0.385	3.13	1.76E-03	1.31E-02	2.235	Upregulated
MAP3K6	72.61	1.042	0.328	3.18	1.47E-03	1.13E-02	2.207	Upregulated
TIAM2	51.90	1.200	0.387	3.10	1.94E-03	1.42E-02	2.203	Upregulated
NPM2	53.65	1.172	0.393	2.98	2.84E-03	1.91E-02	2.080	Upregulated
GLIPR1	40.92	1.270	0.435	2.92	3.52E-03	2.29E-02	2.075	Upregulated
NCF2	27.44	1.479	0.540	2.74	6.14E-03	3.53E-02	2.073	Upregulated
FGF19	47.41	1.220	0.415	2.94	3.29E-03	2.16E-02	2.065	Upregulated
LOC100126784	44.16	1.263	0.441	2.86	4.19E-03	2.62E-02	2.024	Upregulated

GLP2R	62.88	1.049	0.355	2.95	3.13E-03	2.07E-02	1.984	Upregulated
HES2	59.89	1.052	0.360	2.92	3.47E-03	2.26E-02	1.954	Upregulated
EPHB6	62.39	1.032	0.353	2.93	3.44E-03	2.24E-02	1.946	Upregulated
ULBP2	39.43	1.237	0.445	2.78	5.39E-03	3.18E-02	1.943	Upregulated
SAMD11	34.94	1.319	0.488	2.71	6.82E-03	3.82E-02	1.937	Upregulated
SLC19A1	42.67	1.192	0.425	2.80	5.04E-03	3.02E-02	1.931	Upregulated
CXCL8	128.73	1.466	0.585	2.50	1.23E-02	6.01E-02	1.907	Upregulated
PCNXL2	40.43	1.203	0.440	2.73	6.29E-03	3.59E-02	1.880	Upregulated
SLC4A11	35.19	1.283	0.483	2.66	7.82E-03	4.26E-02	1.878	Upregulated
ARID3A	40.67	1.216	0.447	2.72	6.59E-03	3.71E-02	1.877	Upregulated
PTRH1	34.18	1.275	0.479	2.66	7.81E-03	4.26E-02	1.872	Upregulated
FAM86EP	36.68	1.224	0.458	2.67	7.47E-03	4.11E-02	1.849	Upregulated
NRP2	25.95	1.368	0.547	2.50	1.23E-02	6.02E-02	1.833	Upregulated
ZWILCH	53.65	1.079	0.391	2.76	5.81E-03	3.38E-02	1.824	Upregulated
RTN4RL1	28.19	1.337	0.530	2.52	1.16E-02	5.75E-02	1.824	Upregulated
DUSP18	46.66	1.115	0.410	2.72	6.57E-03	3.70E-02	1.814	Upregulated
FGF21	27.44	1.347	0.552	2.44	1.47E-02	6.86E-02	1.780	Upregulated
FAM129A	39.18	1.180	0.452	2.61	8.96E-03	4.72E-02	1.775	Upregulated
C1orf111	38.18	1.171	0.458	2.55	1.06E-02	5.38E-02	1.727	Upregulated
IQCA1	36.68	1.178	0.465	2.53	1.14E-02	5.67E-02	1.715	Upregulated
NAV1	43.42	1.111	0.429	2.59	9.55E-03	4.95E-02	1.714	Upregulated
TIGD3	26.20	1.253	0.540	2.32	2.04E-02	8.74E-02	1.640	Upregulated
CHST15	40.93	1.104	0.445	2.48	1.31E-02	6.28E-02	1.633	Upregulated
MAP4K2	39.93	1.093	0.439	2.49	1.29E-02	6.20E-02	1.629	Upregulated
TCEAL3	33.19	1.163	0.483	2.41	1.60E-02	7.29E-02	1.627	Upregulated
NPL	29.19	1.167	0.514	2.27	2.31E-02	9.56E-02	1.550	Upregulated
CERCAM	42.67	1.035	0.431	2.41	1.62E-02	7.34E-02	1.536	Upregulated
FICD	42.93	1.048	0.438	2.39	1.68E-02	7.55E-02	1.535	Upregulated
IGFLR1	33.69	1.095	0.477	2.29	2.18E-02	9.17E-02	1.508	Upregulated
ANKRD1	2465.17	1.030	0.446	2.31	2.10E-02	8.91E-02	1.471	Upregulated

Factor name	baseMean	log2FoldChange	lfcSE	stat	pvalue	padj	distance	significance
COL6A2	312.77	-3.593	0.215	-16.70	1.28E-62	1.95E-59	58.819	Downregulated
ITIH3	4272.13	-1.445	0.093	-15.61	6.23E-55	7.62E-52	51.139	Downregulated
FADS1	12316.26	-1.278	0.087	-14.75	3.00E-49	2.44E-46	45.630	Downregulated
TTR	45920.62	-1.090	0.074	-14.73	3.86E-49	2.95E-46	45.543	Downregulated
CYP4F2	1809.39	-1.202	0.095	-12.71	4.97E-37	2.76E-34	33.580	Downregulated
FER1L4	880.33	-1.764	0.148	-11.91	1.03E-32	4.34E-30	29.415	Downregulated

UCHL1	683.26	-1.499	0.128	-11.75	6.85E-32	2.70E-29	28.608	Downregulated
MAP3K14	890.46	-1.295	0.118	-10.97	5.48E-28	1.67E-25	24.810	Downregulated
BIRC3	2340.63	-1.313	0.122	-10.76	5.10E-27	1.45E-24	23.875	Downregulated
MTRNR2L2	4266.49	-1.015	0.095	-10.72	8.39E-27	2.28E-24	23.664	Downregulated
NQO1	2789.02	-1.091	0.103	-10.60	3.13E-26	7.98E-24	23.124	Downregulated
PNN	1244.09	-1.190	0.113	-10.55	4.99E-26	1.17E-23	22.962	Downregulated
MTRNR2L8	2408.68	-1.052	0.101	-10.46	1.36E-25	3.08E-23	22.536	Downregulated
MTRNR2L9	2479.24	-1.161	0.111	-10.44	1.60E-25	3.55E-23	22.480	Downregulated
SLCO1B1	488.95	-1.523	0.147	-10.38	2.94E-25	6.31E-23	22.252	Downregulated
XPNPEP2	453.75	-1.534	0.149	-10.31	6.04E-25	1.27E-22	21.949	Downregulated
COL6A1	741.22	-1.461	0.142	-10.30	7.09E-25	1.47E-22	21.882	Downregulated
ALDH1L1	3073.16	-1.029	0.100	-10.25	1.19E-24	2.29E-22	21.664	Downregulated
CYP2D6	462.52	-1.588	0.160	-9.94	2.74E-23	4.65E-21	20.395	Downregulated
CYP1A2	412.79	-1.488	0.153	-9.70	3.13E-22	4.78E-20	19.378	Downregulated
COL7A1	308.46	-1.766	0.201	-8.81	1.29E-18	1.50E-16	15.921	Downregulated
CYP7A1	68.45	-4.725	0.548	-8.63	6.33E-18	7.23E-16	15.861	Downregulated
VCAM1	78.94	-3.365	0.391	-8.60	8.16E-18	8.98E-16	15.418	Downregulated
PDE4C	235.76	-1.889	0.221	-8.53	1.49E-17	1.62E-15	14.910	Downregulated
NUDT4P2	136.42	-10.538	1.459	-7.22	5.19E-13	3.39E-11	14.855	Downregulated
ADH1A	474.00	-1.378	0.162	-8.52	1.55E-17	1.67E-15	14.841	Downregulated
VAMP1	457.53	-1.464	0.174	-8.43	3.42E-17	3.57E-15	14.521	Downregulated
MIR210HG	289.92	-1.684	0.200	-8.41	4.21E-17	4.32E-15	14.462	Downregulated
ZBED6CL	443.25	-1.293	0.154	-8.38	5.26E-17	5.36E-15	14.330	Downregulated
C10orf32-ASMT	288.44	-1.586	0.190	-8.35	6.62E-17	6.57E-15	14.270	Downregulated
GREM2	492.42	-1.189	0.144	-8.26	1.40E-16	1.36E-14	13.918	Downregulated
OLFML1	516.66	-1.165	0.143	-8.16	3.25E-16	3.05E-14	13.565	Downregulated
CYP4F12	494.23	-1.355	0.169	-8.00	1.28E-15	1.11E-13	13.025	Downregulated
SLCO4C1	698.17	-1.031	0.129	-8.00	1.21E-15	1.05E-13	13.018	Downregulated
LINC01089	108.66	-2.385	0.301	-7.93	2.14E-15	1.83E-13	12.958	Downregulated
SRD5A2	224.50	-1.427	0.198	-7.22	5.14E-13	3.38E-11	10.568	Downregulated
GOLGA8A	155.08	-1.744	0.242	-7.20	6.16E-13	3.96E-11	10.547	Downregulated
CAPN10-AS1	98.91	-2.197	0.310	-7.09	1.38E-12	8.46E-11	10.310	Downregulated
LOC730101	422.30	-1.267	0.178	-7.14	9.61E-13	6.03E-11	10.298	Downregulated
CSAD	534.93	-1.237	0.173	-7.13	1.02E-12	6.33E-11	10.273	Downregulated
IL17RB	483.91	-1.009	0.143	-7.06	1.67E-12	9.98E-11	10.052	Downregulated
ZNF292	553.80	-1.021	0.146	-7.00	2.55E-12	1.48E-10	9.883	Downregulated
SNHG3	198.78	-1.455	0.210	-6.94	3.80E-12	2.14E-10	9.779	Downregulated
HLA-G	93.90	-2.182	0.321	-6.79	1.12E-11	5.86E-10	9.486	Downregulated

PDK1	263.43	-1.500	0.222	-6.74	1.57E-11	8.15E-10	9.212	Downregulated
PRLR	128.13	-1.826	0.277	-6.60	4.17E-11	1.97E-09	8.895	Downregulated
PMM1	292.16	-1.167	0.177	-6.59	4.41E-11	2.07E-09	8.761	Downregulated
FBXL19-AS1	412.27	-1.043	0.160	-6.50	7.85E-11	3.50E-09	8.520	Downregulated
LINC00528	53.95	-2.750	0.433	-6.35	2.13E-10	8.85E-09	8.510	Downregulated
HERC5	225.50	-1.263	0.197	-6.41	1.50E-10	6.39E-09	8.291	Downregulated
CYP2A6	282.17	-1.135	0.177	-6.40	1.55E-10	6.55E-09	8.262	Downregulated
SLC27A5	299.94	-1.302	0.209	-6.22	4.98E-10	1.99E-08	7.811	Downregulated
ALOX12P2	156.58	-1.453	0.237	-6.13	8.84E-10	3.40E-08	7.608	Downregulated
LOC153910	89.40	-1.918	0.318	-6.04	1.54E-09	5.64E-08	7.498	Downregulated
FETUB	227.24	-1.187	0.195	-6.09	1.13E-09	4.29E-08	7.463	Downregulated
SLC1A2	46.47	-2.828	0.486	-5.82	5.91E-09	1.96E-07	7.279	Downregulated
CXCL10	54.20	-2.431	0.417	-5.83	5.51E-09	1.85E-07	7.159	Downregulated
LINC01311	53.20	-2.399	0.418	-5.75	9.15E-09	2.93E-07	6.960	Downregulated
PFKFB4	93.65	-1.955	0.339	-5.77	7.93E-09	2.57E-07	6.874	Downregulated
PPFIA4	35.22	-3.184	0.578	-5.51	3.53E-08	1.00E-06	6.792	Downregulated
NUSAP1	117.62	-1.535	0.267	-5.76	8.53E-09	2.75E-07	6.739	Downregulated
AKR1C8P	119.88	-1.572	0.274	-5.74	9.34E-09	2.98E-07	6.712	Downregulated
SSUH2	69.69	-2.129	0.376	-5.67	1.45E-08	4.44E-07	6.700	Downregulated
HNF1A-AS1	116.65	-1.842	0.323	-5.70	1.23E-08	3.83E-07	6.676	Downregulated
PPP1R3E	108.39	-1.596	0.284	-5.61	2.00E-08	5.91E-07	6.430	Downregulated
GPR88	74.18	-1.925	0.348	-5.52	3.30E-08	9.45E-07	6.325	Downregulated
INO80B	254.97	-1.121	0.202	-5.54	3.05E-08	8.84E-07	6.157	Downregulated
LOC729603	163.83	-1.319	0.239	-5.51	3.50E-08	9.94E-07	6.146	Downregulated
ALDOC	1169.63	-1.422	0.259	-5.49	4.09E-08	1.15E-06	6.108	Downregulated
THUMPD3-AS1	150.84	-1.331	0.243	-5.47	4.50E-08	1.24E-06	6.054	Downregulated
HMGCS2	4954.08	-1.220	0.225	-5.43	5.78E-08	1.58E-06	5.929	Downregulated
FAM151A	189.52	-1.195	0.222	-5.37	7.77E-08	2.04E-06	5.815	Downregulated
DSCAML1	27.49	-3.325	0.682	-4.88	1.08E-06	2.20E-05	5.723	Downregulated
MMP3	52.20	-2.127	0.410	-5.19	2.15E-07	5.13E-06	5.701	Downregulated
DCDC5	69.19	-1.888	0.362	-5.21	1.88E-07	4.56E-06	5.665	Downregulated
APBB3	206.75	-1.074	0.202	-5.31	1.10E-07	2.77E-06	5.660	Downregulated
ABCB11	161.83	-1.251	0.237	-5.27	1.36E-07	3.37E-06	5.614	Downregulated
THRSP	855.15	-1.428	0.272	-5.24	1.58E-07	3.83E-06	5.602	Downregulated
KMT2E-AS1	57.20	-1.995	0.389	-5.13	2.89E-07	6.73E-06	5.544	Downregulated
ZNF436-AS1	84.18	-1.758	0.341	-5.16	2.47E-07	5.87E-06	5.519	Downregulated
ASIC3	61.94	-1.917	0.374	-5.13	2.88E-07	6.72E-06	5.517	Downregulated
ADAMTS17	125.86	-1.339	0.258	-5.19	2.11E-07	5.06E-06	5.462	Downregulated

CYP17A1	24.98	-3.173	0.668	-4.75	2.03E-06	3.80E-05	5.441	Downregulated
HSD17B14	82.41	-1.623	0.317	-5.11	3.19E-07	7.37E-06	5.383	Downregulated
NEB	1239.74	-2.058	0.413	-4.98	6.29E-07	1.35E-05	5.286	Downregulated
CYP2B7P	99.89	-1.493	0.294	-5.07	3.98E-07	9.04E-06	5.260	Downregulated
CDCA2	134.08	-1.465	0.290	-5.05	4.37E-07	9.85E-06	5.217	Downregulated
NFYC-AS1	56.95	-1.948	0.393	-4.96	7.01E-07	1.49E-05	5.204	Downregulated
FASN	14540.09	-1.386	0.275	-5.04	4.59E-07	1.02E-05	5.179	Downregulated
OXER1	271.70	-1.085	0.214	-5.07	3.98E-07	9.04E-06	5.159	Downregulated
APOM	185.53	-1.079	0.214	-5.05	4.38E-07	9.85E-06	5.122	Downregulated
C12orf76	167.06	-1.131	0.224	-5.04	4.55E-07	1.02E-05	5.119	Downregulated
BNIP3	4825.32	-1.285	0.256	-5.01	5.44E-07	1.19E-05	5.091	Downregulated
RNPC3	146.34	-1.199	0.244	-4.92	8.66E-07	1.82E-05	4.890	Downregulated
TRIM31	88.90	-1.502	0.309	-4.86	1.15E-06	2.33E-05	4.871	Downregulated
TIGD1	54.94	-1.922	0.402	-4.78	1.80E-06	3.43E-05	4.861	Downregulated
RTN1	151.83	-1.173	0.242	-4.85	1.21E-06	2.42E-05	4.762	Downregulated
C8orf44-SGK3	27.98	-4.422	1.527	-2.90	3.78E-03	2.42E-02	4.709	Downregulated
GSTA2	1666.15	-1.183	0.246	-4.82	1.46E-06	2.87E-05	4.694	Downregulated
MYOM1	180.05	-1.088	0.225	-4.83	1.38E-06	2.72E-05	4.693	Downregulated
NEAT1	6897.57	-1.305	0.274	-4.77	1.86E-06	3.53E-05	4.639	Downregulated
SPATA21	26.23	-2.703	0.617	-4.38	1.19E-05	1.87E-04	4.605	Downregulated
SNHG19	69.18	-1.679	0.359	-4.68	2.94E-06	5.32E-05	4.592	Downregulated
AKR1D1	157.06	-1.114	0.234	-4.76	1.89E-06	3.60E-05	4.582	Downregulated
SPRY1	82.41	-1.484	0.318	-4.67	3.03E-06	5.46E-05	4.514	Downregulated
TENM1	132.85	-1.207	0.257	-4.69	2.69E-06	4.90E-05	4.476	Downregulated
TSSK3	122.88	-1.376	0.299	-4.61	4.07E-06	7.08E-05	4.372	Downregulated
FST	180.02	-1.097	0.238	-4.60	4.16E-06	7.21E-05	4.285	Downregulated
MSC	58.68	-1.713	0.389	-4.41	1.04E-05	1.65E-04	4.153	Downregulated
RAB37	81.66	-1.400	0.318	-4.39	1.11E-05	1.75E-04	4.010	Downregulated
CBLN3	44.70	-1.894	0.446	-4.25	2.18E-05	3.16E-04	3.980	Downregulated
DLG4	123.36	-1.145	0.262	-4.38	1.20E-05	1.87E-04	3.900	Downregulated
ADH1B	1312.88	-1.942	0.466	-4.17	3.03E-05	4.21E-04	3.895	Downregulated
MDM4	125.35	-1.112	0.256	-4.34	1.41E-05	2.18E-04	3.827	Downregulated
IGF1	94.64	-1.256	0.292	-4.30	1.74E-05	2.63E-04	3.794	Downregulated
C1orf220	62.20	-1.658	0.401	-4.14	3.49E-05	4.77E-04	3.712	Downregulated
LDLRAD1	33.97	-2.016	0.502	-4.01	6.00E-05	7.64E-04	3.712	Downregulated
VWCE	2164.32	-1.211	0.285	-4.25	2.16E-05	3.13E-04	3.708	Downregulated
LINC00115	38.71	-1.889	0.466	-4.06	4.99E-05	6.54E-04	3.703	Downregulated
ADH1C	102.90	-1.406	0.336	-4.19	2.79E-05	3.91E-04	3.687	Downregulated

CCDC183	138.59	-1.085	0.255	-4.26	2.05E-05	3.01E-04	3.685	Downregulated
LOC644656	33.96	-2.016	0.505	-3.99	6.64E-05	8.33E-04	3.680	Downregulated
MALAT1	1072.63	-1.290	0.309	-4.17	3.01E-05	4.19E-04	3.616	Downregulated
LOC101928505	90.14	-1.250	0.300	-4.17	3.01E-05	4.19E-04	3.602	Downregulated
NIPBL-AS1	94.64	-1.220	0.292	-4.17	3.03E-05	4.21E-04	3.589	Downregulated
STX16-NPEPL1	158.33	-1.184	0.284	-4.17	3.08E-05	4.27E-04	3.572	Downregulated
SLC23A1	118.61	-1.090	0.263	-4.15	3.34E-05	4.59E-04	3.512	Downregulated
LINC01004	94.15	-1.301	0.317	-4.10	4.19E-05	5.62E-04	3.501	Downregulated
FGF11	81.15	-1.432	0.354	-4.04	5.34E-05	6.92E-04	3.469	Downregulated
CYP2D7	43.71	-1.758	0.450	-3.90	9.56E-05	1.14E-03	3.427	Downregulated
TTLL7	105.12	-1.149	0.283	-4.06	4.85E-05	6.37E-04	3.396	Downregulated
HIST1H4E	49.20	-1.677	0.433	-3.87	1.07E-04	1.26E-03	3.350	Downregulated
LPPR1	41.21	-1.743	0.456	-3.82	1.32E-04	1.51E-03	3.315	Downregulated
ATP1A2	66.69	-1.440	0.370	-3.89	9.95E-05	1.19E-03	3.261	Downregulated
DKFZP434I0714	50.44	-1.531	0.400	-3.83	1.30E-04	1.49E-03	3.215	Downregulated
LOC646471	119.62	-1.122	0.285	-3.94	8.06E-05	9.82E-04	3.210	Downregulated
SPOCK1	123.35	-1.025	0.259	-3.96	7.52E-05	9.25E-04	3.202	Downregulated
ARNT2	29.47	-1.972	0.545	-3.62	2.93E-04	3.00E-03	3.202	Downregulated
MIR17HG	27.22	-1.986	0.557	-3.57	3.58E-04	3.56E-03	3.153	Downregulated
TENM2	99.38	-1.113	0.291	-3.82	1.34E-04	1.53E-03	3.028	Downregulated
ASPA	43.20	-1.599	0.441	-3.63	2.85E-04	2.93E-03	2.996	Downregulated
APOF	116.37	-1.064	0.280	-3.80	1.43E-04	1.62E-03	2.987	Downregulated
GPRIN3	35.21	-1.713	0.481	-3.56	3.69E-04	3.64E-03	2.980	Downregulated
UNC13D	48.46	-1.607	0.446	-3.61	3.11E-04	3.15E-03	2.973	Downregulated
MIR6723	39.21	-1.650	0.467	-3.53	4.14E-04	4.02E-03	2.909	Downregulated
NDRG1	11395.50	-1.430	0.398	-3.59	3.26E-04	3.28E-03	2.866	Downregulated
HEXA-AS1	28.47	-1.835	0.544	-3.37	7.46E-04	6.51E-03	2.854	Downregulated
HSD17B7P2	40.46	-1.564	0.449	-3.49	4.88E-04	4.60E-03	2.812	Downregulated
STX1B	104.12	-1.018	0.276	-3.68	2.29E-04	2.45E-03	2.803	Downregulated
TRPV3	74.92	-1.202	0.335	-3.59	3.27E-04	3.29E-03	2.759	Downregulated
HK2	91.40	-2.008	0.641	-3.13	1.73E-03	1.30E-02	2.756	Downregulated
TLR9	37.21	-1.601	0.471	-3.40	6.73E-04	5.97E-03	2.740	Downregulated
LINC01558	25.97	-1.819	0.563	-3.23	1.22E-03	9.77E-03	2.711	Downregulated
CA9	377.83	-2.439	1.037	-2.35	1.86E-02	8.17E-02	2.670	Downregulated
RDH16	46.20	-1.435	0.421	-3.41	6.54E-04	5.83E-03	2.655	Downregulated
PNCK	41.20	-2.356	0.947	-2.49	1.28E-02	6.20E-02	2.648	Downregulated
SNHG21	31.73	-1.760	0.553	-3.18	1.46E-03	1.13E-02	2.625	Downregulated
SNHG20	60.68	-1.279	0.371	-3.44	5.72E-04	5.21E-03	2.617	Downregulated

PCED1B	66.68	-1.202	0.349	-3.45	5.67E-04	5.19E-03	2.582	Downregulated
SLC25A27	102.14	-1.121	0.323	-3.47	5.18E-04	4.83E-03	2.573	Downregulated
SPARCL1	32.71	-1.632	0.509	-3.20	1.35E-03	1.06E-02	2.563	Downregulated
APOC4-APOC2	29.47	-1.687	0.537	-3.14	1.68E-03	1.27E-02	2.539	Downregulated
MSC-AS1	30.71	-1.635	0.519	-3.15	1.65E-03	1.25E-02	2.510	Downregulated
LOC100128398	67.43	-1.200	0.356	-3.37	7.43E-04	6.49E-03	2.495	Downregulated
LOC171391	93.64	-1.003	0.292	-3.43	6.03E-04	5.45E-03	2.476	Downregulated
DNAJC3-AS1	56.44	-1.249	0.375	-3.33	8.66E-04	7.32E-03	2.474	Downregulated
C15orf48	120.10	-1.723	0.583	-2.95	3.13E-03	2.07E-02	2.409	Downregulated
SNHG10	39.46	-1.468	0.467	-3.15	1.66E-03	1.25E-02	2.403	Downregulated
COL16A1	109.62	-1.007	0.300	-3.36	7.91E-04	6.79E-03	2.391	Downregulated
TMCO6	83.40	-1.029	0.309	-3.33	8.57E-04	7.26E-03	2.374	Downregulated
EFNA1	1404.95	-1.323	0.415	-3.19	1.43E-03	1.10E-02	2.362	Downregulated
CAPN3	106.14	-1.013	0.304	-3.33	8.73E-04	7.36E-03	2.361	Downregulated
PTPRH	67.41	-1.199	0.372	-3.22	1.27E-03	1.01E-02	2.329	Downregulated
ALOX15B	52.94	-1.244	0.389	-3.20	1.39E-03	1.08E-02	2.326	Downregulated
RGAG1	33.96	-1.477	0.487	-3.03	2.43E-03	1.70E-02	2.305	Downregulated
EXPH5	63.68	-1.132	0.351	-3.22	1.28E-03	1.01E-02	2.293	Downregulated
CEMP1	107.87	-1.032	0.317	-3.26	1.11E-03	8.97E-03	2.293	Downregulated
DIRAS2	44.95	-1.302	0.417	-3.13	1.77E-03	1.32E-02	2.287	Downregulated
LOC100506127	54.95	-1.288	0.412	-3.12	1.78E-03	1.32E-02	2.278	Downregulated
COLCA1	41.96	-1.410	0.463	-3.04	2.35E-03	1.65E-02	2.273	Downregulated
SLC6A13	37.71	-1.426	0.483	-2.96	3.12E-03	2.07E-02	2.207	Downregulated
IGSF1	54.93	-1.163	0.379	-3.07	2.14E-03	1.53E-02	2.155	Downregulated
NRN1	36.21	-1.395	0.481	-2.90	3.75E-03	2.40E-02	2.137	Downregulated
LHX4-AS1	27.48	-1.623	0.633	-2.56	1.03E-02	5.26E-02	2.066	Downregulated
GP1BA	44.20	-1.229	0.425	-2.89	3.85E-03	2.46E-02	2.025	Downregulated
UTS2B	38.21	-1.311	0.466	-2.82	4.86E-03	2.95E-02	2.015	Downregulated
LBHD1	69.93	-1.057	0.360	-2.94	3.31E-03	2.17E-02	1.971	Downregulated
NEURL2	33.21	-1.325	0.485	-2.73	6.32E-03	3.60E-02	1.960	Downregulated
LOC440434	70.92	-1.041	0.357	-2.91	3.57E-03	2.31E-02	1.939	Downregulated
ZNF674-AS1	35.21	-1.289	0.472	-2.73	6.37E-03	3.61E-02	1.934	Downregulated
C9orf173-AS1	37.46	-1.365	0.515	-2.65	8.00E-03	4.34E-02	1.929	Downregulated
SLC22A25	35.46	-1.303	0.484	-2.70	7.04E-03	3.91E-02	1.918	Downregulated
CFAP57	26.72	-1.430	0.559	-2.56	1.05E-02	5.32E-02	1.915	Downregulated
DUSP5P1	49.45	-1.136	0.403	-2.82	4.78E-03	2.90E-02	1.911	Downregulated
APLN	36.45	-1.263	0.470	-2.69	7.25E-03	4.00E-02	1.884	Downregulated
PAXIP1-AS1	65.43	-1.011	0.353	-2.86	4.20E-03	2.63E-02	1.876	Downregulated

GUCY2D	33.96	-1.266	0.480	-2.64	8.38E-03	4.50E-02	1.848	Downregulated
EGFR-AS1	182.01	-1.270	0.487	-2.61	9.12E-03	4.79E-02	1.831	Downregulated
OXT	58.18	-1.021	0.365	-2.80	5.18E-03	3.08E-02	1.824	Downregulated
SKAP1	34.21	-1.280	0.499	-2.57	1.03E-02	5.24E-02	1.811	Downregulated
NEFL	31.21	-1.308	0.516	-2.53	1.13E-02	5.64E-02	1.809	Downregulated
AQP7	50.19	-1.101	0.405	-2.72	6.61E-03	3.72E-02	1.804	Downregulated
IL1RN	53.93	-1.033	0.377	-2.74	6.11E-03	3.52E-02	1.783	Downregulated
ANXA10	35.71	-1.220	0.473	-2.58	9.87E-03	5.09E-02	1.778	Downregulated
TMEM44-AS1	55.93	-1.022	0.376	-2.72	6.53E-03	3.68E-02	1.761	Downregulated
B4GALNT1	42.20	-1.133	0.433	-2.62	8.86E-03	4.69E-02	1.746	Downregulated
FZD7	54.44	-1.023	0.379	-2.70	6.91E-03	3.85E-02	1.746	Downregulated
SCML4	39.95	-1.140	0.437	-2.61	9.13E-03	4.80E-02	1.743	Downregulated
RNF144A	54.43	-1.053	0.395	-2.67	7.63E-03	4.18E-02	1.735	Downregulated
CNTNAP2	28.22	-1.280	0.526	-2.43	1.49E-02	6.96E-02	1.726	Downregulated
EGLN3	1761.91	-1.109	0.426	-2.60	9.19E-03	4.82E-02	1.722	Downregulated
LOC100289230	27.47	-1.288	0.542	-2.38	1.74E-02	7.74E-02	1.701	Downregulated
SPAG4	227.70	-1.096	0.426	-2.57	1.00E-02	5.15E-02	1.691	Downregulated
STXBP6	38.45	-1.234	0.508	-2.43	1.52E-02	7.03E-02	1.689	Downregulated
KIAA1875	34.21	-1.180	0.477	-2.48	1.33E-02	6.37E-02	1.680	Downregulated
ZNF471	36.20	-1.155	0.465	-2.48	1.30E-02	6.25E-02	1.668	Downregulated
NDUFA4L2	2936.87	-1.091	0.432	-2.53	1.15E-02	5.72E-02	1.654	Downregulated
CIT	49.70	-1.047	0.411	-2.54	1.09E-02	5.50E-02	1.638	Downregulated
LOC100272217	32.46	-1.173	0.490	-2.39	1.68E-02	7.54E-02	1.623	Downregulated
TREX1	46.20	-1.062	0.424	-2.50	1.24E-02	6.03E-02	1.617	Downregulated
HERC2P7	37.96	-1.118	0.466	-2.40	1.65E-02	7.46E-02	1.587	Downregulated
C8orf46	42.70	-1.041	0.425	-2.45	1.43E-02	6.74E-02	1.567	Downregulated
LINC00641	37.22	-1.166	0.506	-2.30	2.12E-02	9.00E-02	1.566	Downregulated
REC8	29.97	-1.168	0.512	-2.28	2.26E-02	9.40E-02	1.556	Downregulated
STAG3L3	34.21	-1.131	0.488	-2.32	2.06E-02	8.78E-02	1.548	Downregulated
SLC25A21-AS1	40.70	-1.056	0.442	-2.39	1.68E-02	7.55E-02	1.541	Downregulated
ECM1	37.95	-1.074	0.454	-2.37	1.80E-02	7.96E-02	1.537	Downregulated
MEX3B	32.96	-1.102	0.481	-2.29	2.20E-02	9.24E-02	1.512	Downregulated
PRRT2	36.46	-1.077	0.478	-2.25	2.42E-02	9.90E-02	1.473	Downregulated
HIST4H4	34.71	-1.065	0.471	-2.26	2.36E-02	9.69E-02	1.471	Downregulated

S2 Table Antibodies used in this study

Abbreviated name	Official name	Company name	Catalog No.	Diluted concentration
Rabbit	Peroxidase AffiniPure Goat Anti-Rabbit IgG (H+L)	Jackson Immuno Research	111-035-003	1:10,000
Mouse	Goat anti-Mouse IgG (H+L) Secondary Antibody, HRP	Thermo Fisher Scientific	31430	1:10,000
HA	Purified anti-HA.11 Epitope Tag Antibody	BioLegend	901501	1:1,000
GAPDH	Anti GAPDH, Monoclonal Antibody, Peroxidase Conjugated	Fujifilm	015-25473	1:10,000
N4BP1	N4BP1 Antibody	Bethyl	A304-628A-T	1:1,000
ZCCHC10	Anti-ZCCHC10 antibody produced in rabbit	Sigma	HPA038944	1:500
HNRNPC	Anti-hnRNP C1 + C2/HNRNPC 抗体 [EP3034Y]	abcam	ab75822	1:1,000
KHNYN	KHNYN Antibody	Fujifilm	NBP2-17041	1:1,000
ZC3H12B	ZC3H12B antibody	Gene Tex	GTX85196	1:1,000
Alexa-488	F(ab')2-Goat anti-Rabbit IgG (H+L) Cross-Adsorbed Secondary Antibody, Alexa Fluor™ Plus 488	Thermo Fisher Scientific	A48282	1:1,000
HA-Magnetic Beads	Anti-HA-tag mAb-Magnetic Beads	Medical & Biological Laboratories	M180-11	25ul/sample
